# Socioeconomic differences in health-care use and outcomes for stroke and ischaemic heart disease in China during 2009–16: a prospective cohort study of 0·5 million adults

**DOI:** 10.1016/S2214-109X(20)30078-4

**Published:** 2020-03-18

**Authors:** Muriel Levy, Yiping Chen, Robert Clarke, Derrick Bennett, Yunlong Tan, Yu Guo, Zheng Bian, Jun Lv, Canqing Yu, Liming Li, Winnie Yip, Zhengming Chen, Borislava Mihaylova

**Affiliations:** a Clinical Trial Service Unit and Epidemiological Studies Unit, Nuffield Department of Population Health, University of Oxford, Oxford, UK; b Health Economics Research Centre, Nuffield Department of Population Health, University of Oxford, Oxford, UK; c Medical Research Council Population Health Research Unit at the University of Oxford, Nuffield Department of Population Health, University of Oxford, Oxford, UK; d Chinese Academy of Medical Sciences, Beijing, China; e Department of Epidemiology and Biostatistics, School of Public Health, Peking University Health Science Centre, Beijing, China; f Harvard T H Chan School of Public Health, Boston, MA, USA; g Institute of Population Health Sciences, Queen Mary University of London, London, UK

## Abstract

**Background:**

China initiated major health-care reforms in 2009 aiming to provide universal health care for all by 2020. However, little is known about trends in health-care use and health outcomes across different socioeconomic groups in the past decade.

**Methods:**

We used data from the China Kadoorie Biobank (CKB), a nationwide prospective cohort study of adults aged 30–79 years in 2004–08, in ten regions (five urban, five rural) in China. Individuals who were alive in 2009 were included in the present study. Data for all admissions were obtained by linkage to electronic hospital records from the health insurance system, and to region-specific disease and death registers. Generalised linear models were used to estimate trends in annual hospital admission rates, 28-day case fatality rates, and mean length of stay for stroke, ischaemic heart disease, and any cause in all relevant individuals.

**Findings:**

512 715 participants were recruited to the CKB between June 25, 2004, and July 15, 2008, 505 995 of whom were still alive on Jan 1, 2009, and contributed to the present study. Among them, we recorded 794 824 hospital admissions (74 313 for stroke, 69 446 for ischaemic heart disease) between 2009 and 2016. After adjustment for demographic, socioeconomic, lifestyle, and morbidity factors, hospitalisation rates increased annually by 3·6% for stroke, 5·4% for ischaemic heart disease, and 4·2% for any cause, between 2009 and 2016. Higher socioeconomic groups had higher hospitalisation rates, but the annual proportional increases were higher in those with lower education or income levels, those enrolled in the urban or rural resident health insurance scheme, and for those in rural areas. Lower socioeconomic groups had higher case fatality rates for stroke and ischaemic heart disease, but greater reductions in case fatality rates than higher socioeconomic groups. By contrast, mean length of stay decreased by around 2% annually for stroke, ischaemic heart disease, and any cause, but decreased to a greater extent in higher than lower socioeconomic groups for stroke and ischaemic heart disease.

**Interpretation:**

Between 2009 and 2016, lower socioeconomic groups in China had greater increases in hospital admission rates and greater reductions in case fatality rates for stroke and ischaemic heart disease. Additional strategies are needed to further reduce socioeconomic differences in health-care use and disease outcomes.

**Funding:**

Wellcome Trust, Medical Research Council, British Heart Foundation, Cancer Research UK, Kadoorie Charitable Foundation, China Ministry of Science and Technology, and Chinese National Natural Science Foundation.

## Introduction

China has seen a rapid demographic and epidemiological transition in recent decades, resulting in an increasing burden of chronic non-communicable diseases and escalating financial pressures on the health-care system.^1^ Cardiovascular diseases, chiefly stroke and ischaemic heart disease, are the leading causes of premature death and permanent disability in China, accounting for about 100 million prevalent cases and 4 million deaths in 2017.^2^ Due to the scarcity of effective primary care and limited implementation of primary and secondary prevention treatments for cardiovascular disease, hospitalisation (referring to admission to hospital) costs for cardiovascular disease have increased substantially in China.^1^, ^3^, ^4^

In response to growing inequalities in health-care use and disease outcomes between urban and rural areas, new health insurance programmes were initiated in China in 2003.^5^ In 2009, the Chinese Government launched a major health-care reform aiming to provide affordable and equal access to high quality basic health services for all by 2020.^6^ The first cycle of this reform (2009–12) included an expansion of health insurance to the entire population and increase in benefits, strengthening of the primary health-care and public health services, and establishment of a national essential medicines system, while the second cycle (2013–16) focused more on improving the efficiency and quality of public hospital services.^6^ However, little is known about trends in health-care use and outcomes for affected individuals with stroke and ischaemic heart disease in urban and rural areas, and in different socioeconomic groups in the past decade.^6^, ^7^

**Research in context**

**Evidence before this study**

We searched for studies investigating trends in hospital use for cardiovascular diseases and any cause, by socioeconomic characteristics in the past decade in China, from Jan 1, 2007, to March 15, 2019, using MEDLINE and Embase. The search strategy included terms related to hospital admissions (“hospitalisation”, “admission”, and “inpatient”), performance measures (“utilisation”, “rate”, “length of stay”, “trends”, “case fatality”, “mortality”, “quality”, “efficiency”, “equity”), and socioeconomic characteristics influencing the use of care (“insurance”, “socioeconomic”, “education”, “occupation”, “income”, “urban”, and “rural”). Multiple possible synonyms and spellings of search terms were also used. We included studies that were based in mainland China and involved hospital admissions for stroke, ischaemic heart disease, or any cause in the general population. Most studies had a cross-sectional or retrospective design, focused on specific regions (eg, low-income regions), populations (urban or rural) or health insurance enrollees (mainly new rural cooperative medical scheme). These studies investigated the effect of health insurance on health-care use or measured inequalities in the use of inpatient or outpatient care for any cause. A few studies explored the disparities in health outcomes and health-care use across health insurance groups, but only among patients with acute myocardial infarction, heart failure, and intracranial haemorrhage. Studies that examined trends in hospital use for stroke and ischaemic heart disease did not include variations by socioeconomic characteristics. Previous studies using data from the 2003, 2008, and 2011 National Health Services Survey and medical records from 11 hospitals nationwide, reported increasing trends in hospitalisation rates for any cause before and after the 2009 health-care reforms. On the basis of data from over 100 tertiary hospitals in 2007–10, it was reported that the number of hospitalisations for stroke increased and in-hospital mortality decreased. The China PEACE study reported increased admission rates for acute myocardial infarction, decreased mean length of stay, and stable adjusted in-hospital mortality in the period 2001–11. A study in Beijing, China, in 2007–12 reported that hospitalisation rates for acute myocardial infarction increased, but mean length of stay and in-hospital mortality decreased.

**Added value of this study**

This is the first large study, to our knowledge, to provide a comprehensive analysis of trends in hospital admissions for common cardiovascular diseases in China between 2009 and 2016, both overall and across different socioeconomic groups. In men and women aged 30–79 years at entry in the China Kadoorie Biobank, after adjusting for demographic, socioeconomic, lifestyle, and morbidity factors, the hospitalisation rates for stroke, ischaemic heart disease, and any cause increased by approximately 4–5% per year in this period. Individuals living in rural areas, with lower education or income levels, and enrolled in the urban or rural resident health insurance scheme had the greatest annual increases in rates of hospitalisation for stroke and ischaemic heart disease, and the greatest reductions in 28-day case fatality rates. The mean length of stay for stroke, ischaemic heart disease, and any cause decreased by about 2% each year and decreased to a greater extent in higher socioeconomic groups during 2009–16.

**Implications of all the available evidence**

The available evidence showed substantial increases in hospital admissions and reductions in case fatality rates and mean length of stay for stroke and ischaemic heart disease in Chinese adults aged 30–90 years between 2009 and 2016. Although socioeconomic inequalities in hospital use persist, improvements in health-care use and health outcomes during this period have been greatest in rural areas and among lower socioeconomic groups. These results should inform strategies for health promotion and equitable distribution of health-care resources. Further improvements in prevention and management of cardiovascular disease are needed to reverse the trends in incidence and achieve further reductions in inequalities in hospital use and outcomes for stroke and ischaemic heart disease.

Previous studies of the burden of stroke and ischaemic heart disease in China have mainly focused on trends in incidence, prevalence, mortality, or disability-adjusted life-years.^3^, ^8^ Most of the available studies of the use of health-care services for stroke and ischaemic heart disease in China have been constrained by short duration of follow-up, restriction to individual hospitals, or inability to study the effects of person-level characteristics on disease outcomes.^5^, ^9^, ^10^, ^11^, ^12^, ^13^, ^14^, ^15^, ^16^ To address these limitations, the aims of the present study were to: (1) examine trends in annual rates of hospitalisations for stroke, ischaemic heart disease, and all causes combined together (any cause) between 2009 and 2016; (2) investigate differences in hospitalisation rates between different socioeconomic groups over this period; and (3) assess whether trends in case fatality rates and mean length of stay for stroke and ischaemic heart disease differ between socioeconomic groups.

## Methods

### Study design and participants

We used data from the China Kadoorie Biobank (CKB), a prospective cohort study of adults, who were recruited from five urban (Qingdao, Harbin, Haikou, Suzhou, Liuzhou) and five rural (Gansu, Sichuan, Henan, Zhejiang, Hunan) areas in China between June 25, 2004, and July 15, 2008.^17^ Official local residential records were used to identify eligible individuals (adults aged 30–79 years). All participants completed an interviewer-administered electronic questionnaire, providing detailed information on demographic and socioeconomic characteristics, medical history, and lifestyle factors (appendix 2 p 5). In addition, physical measurements were recorded, and a blood sample was collected for long-term storage. Individuals who were alive on Jan 1, 2009 contributed to the present study. All participants in the CKB provided written informed consent for participation and long-term follow-up for health status by electronic linkage to relevant medical records. Prior international, national, and local ethical approvals were obtained. The protocol for the CKB has been previously published.^17^, ^18^

### Procedures

Data for all hospital admissions were obtained by ongoing linkage via unique national identification number, to electronic hospital records from the nationwide health insurance system (which has >98% coverage across the ten study areas), to registers of stroke and ischaemic heart disease established by CKB's regional centres, and to death registers monitored through China's Disease Surveillance Points and Information System (appendix 2 p 5). Diagnoses associated with each hospital admission were reviewed, integrated centrally and standardised using the International Classification of Diseases 10th revision (ICD-10) codes using a bespoke programme. Hospital admissions were identified using ICD-10 codes I60-I61 and I63-I64 for stroke, and I20-I25 for ischaemic heart disease. Overall, 92% of reported incident strokes had their diagnoses confirmed by CT or MRI and 97% of incident ischaemic heart disease cases had electrocardiogram reports. Hospital admissions included all first and subsequent admissions occurring in public and private hospitals across all hospital tiers.

### Statistical analysis

The study outcomes were the annual number of hospital admissions, 28-day case fatality, and length of stay of admissions for stroke, ischaemic heart disease, and any cause. Since recruitment to the CKB was completed on July 15, 2008, the present analyses were restricted to all hospital admissions occurring between Jan 1, 2009, and Dec 31, 2016. We defined the length of stay as the difference in days between the admission date and the discharge date of the hospitalisation or date of death, when relevant. Day case admissions (ie, participant admitted and discharged on the same day) were counted as having a length of stay of 0·5 days. Missing values for length of stay were imputed using multiple imputation (appendix 2 p 6). The 28-day case fatality was defined as death within 28 days from the first-ever event for stroke or ischaemic heart disease for participants without history of stroke, transient ischaemic attack, and ischaemic heart disease before 2009.

Annual rates of hospital admissions were estimated using generalised linear models (GLM) with log-link function and negative binomial distribution. Annual 28-day case fatality rates were estimated using GLM with logit link function and binomial distribution. Annual mean length of stay per admission was estimated using GLM with log link function and gamma distribution (appendix 2 p 6).^19^

We examined hospitalisation rates, case fatality rates, and mean length of stay for stroke, ischaemic heart disease, and any cause, overall and by the following factors: urban and rural area of residence, highest level of education attained (no formal school, primary or middle school, high school or above), annual household income in renminbi (<¥10 000, ¥10 000–19 999, ¥20 000–34 999, ≥¥35 000), and health insurance type (urban employee basic medical insurance [UEBMI], urban resident basic medical insurance [URBMI], or new rural cooperative medical scheme [NRCMS]: appendix 2 p 8). The annual rates of hospitalisation, 28-day case fatality, and mean length of stay, and their annual trends (percentage change per calendar year) were estimated with adjustment for annually updated age (ie, continuous age, age-squared, age-cubed), sex, and region (ie, minimally adjusted models). In further analyses, socioeconomic, lifestyle, and morbidity factors were also included (ie, fully adjusted models), to evaluate annual rates and trends most indicative of changes in the health-care system, including changes in the organisation and delivery of health care. Socioeconomic factors included marital status, household size, highest level of education attained, annual household income, and health insurance type. Lifestyle factors included smoking and alcohol consumption, body-mass index, physical activity assessed using metabolic equivalents of task, and self-rated health status. Classification of socioeconomic (except health insurance type) and lifestyle factors was restricted to information collected at entry into the study (2004–08). Morbidity factors included history of self-reported doctor-diagnosed diseases at entry into CKB, with previous history of major diseases (cerebrovascular disease, ischaemic heart disease, malignant neoplasms, respiratory diseases, infectious and parasitic diseases, diabetes, chronic kidney disease, and tuberculosis) updated annually at the start of each year from recruitment to Dec 31, 2016, using linked CKB data. Additional adjustments were made for stroke type (ischaemic, haemorrhagic, or unspecified) and ischaemic heart disease type (acute myocardial infarction or other ischaemic heart disease) in analyses of case fatality and mean length of stay.

Data for health insurance types for each participant were identified annually in 2012–16, but were unavailable for the years 2009–11; the missing data for health insurance type were imputed based on the insurance scheme in which participants were enrolled in 2012 (appendix 2 pp 6–7). Health insurance types were classified as UEBMI, URBMI, NRCMS, or uninsured. These health insurance schemes differed in their target population, administration, source of funding, and benefits, with UEBMI providing the most comprehensive coverage (appendix 2 p 8).^6^ In the analyses, URBMI and NRCMS were combined, as they provided similar benefits, and in four of the ten CKB regions, the two schemes merged into a single scheme from 2012–13 onwards. Participants who were uninsured were excluded from analyses by health insurance type because of the small number of cases. Models for trends by urban or rural areas did not include adjustments for health insurance type, because of substantial correlation between these two factors. Heterogeneity or, if relevant (ie, more than two ordered categories), trends in annual rates of hospitalisation, case fatalities, and mean length of stay between categories of participants by urban and rural areas, education, income, and health insurance type were assessed using χ^2^ tests for heterogeneity or trend. Standardised predicted admission rates (per 1000 person-years), case fatality rates (per 100 events), and mean length of stay (in days) for each calendar year of follow-up were derived using characteristics of all CKB study participants in 2009, except for models by urban and rural area. As each CKB region contributed data for only urban or only rural area, the estimates for urban and rural areas, in which adjustment for region were used, were standardised using the separate CKB populations in urban and rural areas in 2009. Annual absolute differences in rates of hospitalisation, case fatality rate, and mean length of stay between the two most extreme groups of each socioeconomic characteristic, and an annual slope index of inequality for each characteristic except urban and rural area, were also estimated, together with a test for linear trend. The slope index of inequality is a weighted measure of inequality that takes into account the entire distribution of each socioeconomic characteristic, rather than only comparing the two most extreme groups.^20^, ^21^ Cluster-adjusted robust SEs were estimated to control for absence of independence between hospital admissions of the same participant during follow-up (appendix 2 p 8).

In sensitivity analyses, hospital admissions were restricted to first-ever admissions for stroke or ischaemic heart disease in the study, and the annual differences in hospitalisation rates, case fatality rates, and mean length of stay, and slope index of inequality between urban and rural area were standardised for the entire CKB study population in 2009, but without adjustment for region. In further analyses, trends in hospitalisation rates, case fatality rates, and mean length of stay were assessed during the two cycles of the health-care reforms (2009–12, 2013–16), by stroke and ischaemic heart disease type, and by age group (<60, 60–69, ≥70 years). All analyses were done using Stata 15 or R 3.6.0.

### Role of the funding source

The funders of the study had no role in study design, data collection, data analysis, data interpretation, or writing of the report. ML, YC, RC, ZC, and BM had full access to all the data in the study and had final responsibility for the decision to submit for publication.

## Results

Enrolment in the CKB took place between June 25, 2004, and July 15, 2008. 1 801 167 eligible individuals were identified, and 512 715 (28%) of those invited responded and were enrolled. Among the 512 715 participants in the CKB, 6469 died and 251 were lost to follow-up before 2009, leaving 505 995 (99%) participants contributing data between Jan 1, 2009, and Dec 31, 2016, in the present analyses. Among the 505 995 participants, the mean age was 54·7 years (SD 10·6) in 2009 and 59·2% (299 793) were women (table). Socioeconomic characteristics varied substantially between the ten regions in China, particularly between the five urban and five rural areas (appendix 2 p 9). The proportion of participants who had completed high school education was higher in those living in urban (n=80 593, 36·0%) than in rural (n=26 164, 9·3%) areas, as was the proportion of participants with an annual household income of ¥35 000 or greater in urban (n=56 974, 25·4%) compared with rural areas (n=34 758, 12·3%). In 2012, 280 967 (56·8%) participants were enrolled in URBMI or NRCMS, 186 369 (37·7%) were enrolled in UEBMI, and only 27 027 (5·5%) were uninsured. Most individuals living in urban areas were enrolled in the UEBMI, while most participants living in rural areas were enrolled in the NRCMS (table). During 3·92 million person-years of follow-up between 2009 and 2016, 794 824 hospital admissions were recorded for 268 070 participants, which included 74 313 (9·3%) admissions for stroke and 69 446 (8·7%) admissions for ischaemic heart disease.

**Table:** Background characteristics in the CKB

		All (N=505 995*)	**Urban (n=224 093)**	**Rural (n=281 902)**
Age,† years		54·7 (10·6)	55·4 (10·9)	54·2 (10·4)
Sex				
	Women	299 793 (59·2%)	133 933 (59·8%)	165 860 (58·8%)
	Men	206 202 (40·8%)	90 160 (40·2%)	116 042 (41·2%)
Medical history				
	Diabetes	15 604 (3·1%)	10 255 (4·6%)	5349 (1·9%)
	Hypertension	58 237 (11·5%)	30 625 (13·7%)	27 612 (9·8%)
	Coronary heart disease	15 018 (3·0%)	10 267 (4·6%)	4751 (1·7%)
	Stroke or transient ischaemic attack	8454 (1·7%)	5081 (2·3%)	3373 (1·2%)
Body-mass index,‡ kg/m^2^				
	Normal, <25·0	338 770 (67·0%)	134 233 (59·9%)	204 537 (72·6%)
	Overweight, 25·0–29·9	146 475 (28·9%)	77 721 (34·7%)	68 754 (24·4%)
	Obese, ≥30·0	20 748 (4·1%)	12 139 (5·4%)	8609 (3·1%)
Physical activity, metabolic equivalent h per day		21·2 (13·9)	18·5 (12·6)	23·3 (14·4)
Systolic blood pressure, mm Hg		130·9 (21·1)	129·2 (21·0)	132·3 (21·1)
Current smoker				
	Men	126 084 (61·1%)	49 517 (54·9%)	76 567 (66·0%)
	Women	6981 (2·3%)	2713 (2·0%)	4268 (2·6%)
Current alcohol drinker§				
	Men	92 090 (44·7%)	45 067 (50·0%)	47 023 (40·5%)
	Women	11 695 (3·9%)	5749 (4·3%)	5946 (3·6%)
Self-reported poor health status¶		51 187 (10·1%)	20 205 (9·0%)	30 982 (11·0%)
Married		459 051 (90·7%)	200 278 (89·4%)	258 773 (91·8%)
Household size		3·8 (1·5)	3·2 (1·3)	4·2 (1·5)
Education				
	No formal school	92 965 (18·4%)	26 434 (11·8%)	66 531 (23·6%)
	Primary or middle school	306 273 (60·5%)	117 066 (52·2%)	189 207 (67·1%)
	High school and above	106 757 (21·1%)	80 593 (36·0%)	26 164 (9·3%)
Household income, ¥ per year				
	<¥10 000	141 424 (28·0%)	30 188 (13·5%)	111 236 (39·5%)
	¥10 000–19 999	147 143 (29·1%)	65 436 (29·2%)	81 707 (29·0%)
	¥20 000–34 999	125 696 (24·8%)	71 495 (31·9%)	54 201 (19·2%)
	≥¥35 000	91 732 (18·1%)	56 974 (25·4%)	34 758 (12·3%)
Health insurance type‖				
	UEBMI	186 369 (37·7%)	158 288 (71·9%)	28 081 (10·2%)
	URBMI or NRCMS**	280 967 (56·8%)	44 505 (20·2%)	236 462 (86·2%)
	Uninsured	27 027 (5·5%)	17 321 (7·9%)	9706 (3·5%)

Data are mean (SD) or n (%). UEBMI=urban employee basic medical insurance. URBMI=urban resident basic medical insurance. NRCMS=new rural cooperative medical scheme. CKB=China Kadoorie Biobank.

* From the 512 715 participants initially recruited in the CKB, 6469 died and 251 were lost to follow-up in 2005–08. In 2009–16, 4500 participants were lost to follow-up.

† Age is for the year 2009. Mean age at entry in CKB was 51·7 years (SD 10·6) for all, 52·8 (10·8) for urban, and 51·2 (10·4) for rural participants.

‡ 2 participants had missing data on body-mass index. Underweight individuals were grouped as “normal”.

§ Regular alcohol drinkers were those who drank alcohol at least monthly.

¶ Self-reported poor health status was defined as a response of “poor” from either excellent, good, fair, or poor by participants when answering the question, “how is your current general health status”.

‖ Information on health insurance type is for the year 2012 (n=494 363), as annual linkage to participants' schemes were only available from 2012 and missing health insurance type in 2009–11 was imputed based on the scheme participants were enrolled on in 2012. Uninsured participants include 307 (1·1%) participants with missing health insurance type.

** In the ten CKB centres, 263 916 (53·4%) individuals were enrolled in NRCMS and 17 038 (3·5%) in URBMI. In urban areas, 27 720 (12·6%) individuals were enrolled in NRCMS, while in rural areas 236 196 (86·1%) were enrolled in NRCMS.

**Figure 1:**
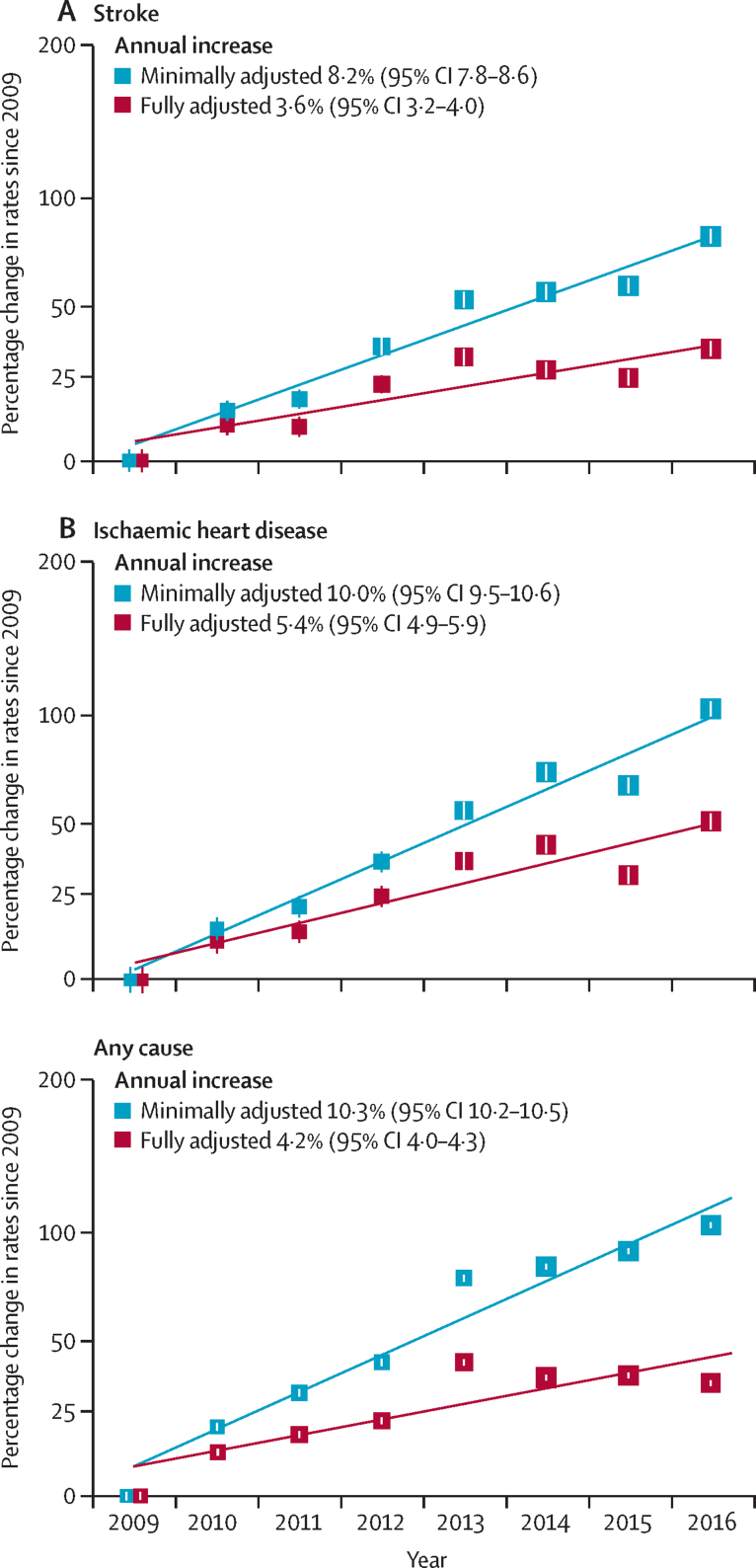
Change in rates of hospitalisation for stroke, ischaemic heart disease, and any cause, since 2009 Generalised linear regression models with negative binomial distribution and log link function were used. The minimally adjusted models included adjustments for age, sex, and region. The fully adjusted models included adjustments for demographic factors (age and sex), socioeconomic factors (marital status, household size, education, income, and health insurance type), lifestyle factors (smoking, alcohol consumption, BMI, physical activity, self-reported health), morbidity factors, and region. Two participants had missing data for BMI. Rates are shown with 95% CIs, which are based on floating absolute risks. The area of each square is inversely proportional to the variance. BMI=body-mass index.

The hospitalisation rates adjusted for age, sex, and region increased by 8·2% for stroke (95% CI 7·8–8·6), 10·0% for ischaemic heart disease (9·5–10·6), and 10·3% for any cause (10·2–10·5) per year. Following further adjustment for individual socioeconomic, lifestyle, and morbidity factors, the corresponding annual increases in hospitalisation rates decreased by about half to 3·6% for stroke (95% CI 3·2–4·0), 5·4% for ischaemic heart disease (4·9–5·9), and 4·2% for any cause (4·0–4·3) (figure 1). The fully adjusted annual hospital admission rates increased from 10·9 (95% CI 10·5–11·2) in 2009 to 14·5 (14·2–14·9) admissions per 1000 person-years in 2016 for stroke; from 9·3 (8·9–9·6) to 14·1 (13·7–14·4) for ischaemic heart disease, and from 112·2 (111·0–113·3) to 151·2 (150·0–152·5) for any cause (appendix 2 p 16). The annual increases in rates of hospitalisation for stroke, ischaemic heart disease, and any cause were higher during the first cycle of the reforms (2009–12) than the second cycle (2013–16). The annual increases in rates of hospitalisation for stroke were 5·7% (95% CI 4·4 to 7·0) in the first cycle versus 0·5% (–0·4 to 1·5) in the second cycle; for ischaemic heart disease were 7·0% (5·4 to 8·5) versus 2·6% (1·5 to 3·6); and for any cause were 5·0% (4·6 to 5·5) versus −0·7% (–1·1 to −0·4; appendix 2 p 10).

**Figure 2:**
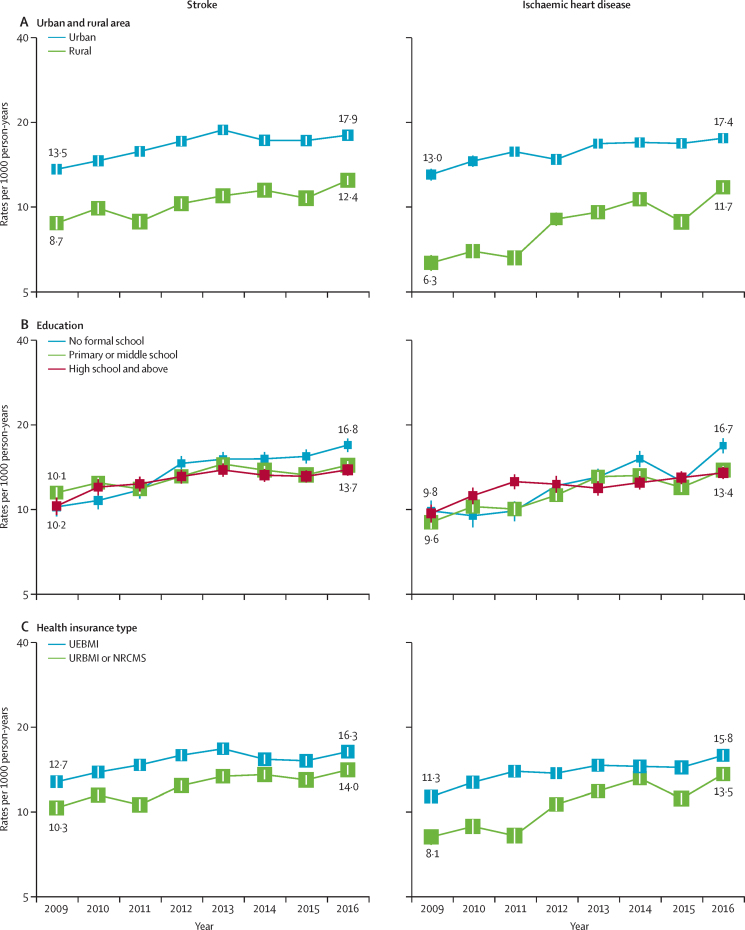
Annual rates of hospitalisation for stroke and ischaemic heart disease, by urban and rural area, education, and health insurance type Generalised linear regression models with negative binomial distribution and log link function were used. Fully adjusted models included adjustments for demographic, socioeconomic, lifestyle, and morbidity factors, as well as region. In analyses by health insurance type, uninsured participants were excluded because of the small number of cases. Rates by education and health insurance type were standardised for the overall CKB participant population in 2009 and rates by urban and rural area were standardised separately for the CKB participant population living in urban or rural areas, in 2009. Rates per 1000 person-years are shown from 2009 to 2016. The area of each square is inversely proportional to the variance. 95% CIs are shown. Two participants had missing data for body-mass index. CKB=China Kadoorie Biobank. UEBMI=urban employee basic medical insurance. URBMI=urban resident basic medical insurance. NRCMS=new rural cooperative medical scheme.

**Figure 3:**
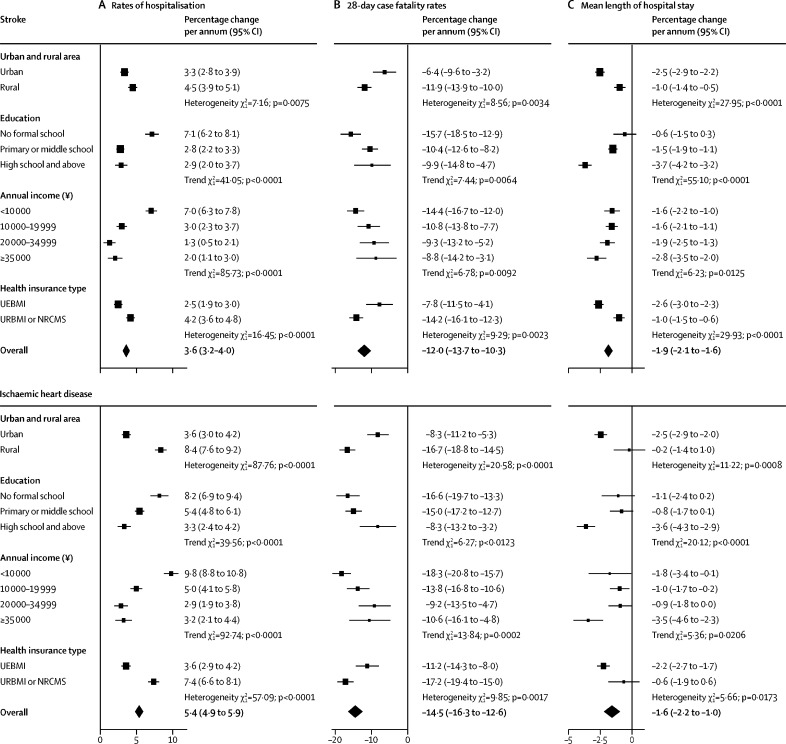
Annual change in rates of hospitalisation, 28-day case fatality, and mean length of hospital stay for stroke and ischaemic heart disease, by urban and rural area, education, income, and health insurance type For rates of hospitalisation: GLM with negative binomial distribution and log link function were used. For 28-day case fatality rates: GLM with binomial distribution and logit link function were used. For mean length of stay: GLM with gamma distribution and log link were used. Fully adjusted models included adjustments for demographic, socioeconomic, lifestyle, and morbidity factors, and region. Models for case fatality rates and mean length of stay also included adjustment for case mix (stroke types: ischaemic, haemorrhagic, and unspecified; and ischaemic heart disease types: acute myocardial infarction and other ischaemic heart disease). In analyses by health insurance type, uninsured participants were excluded due to the small number of cases. Two participants had missing data for BMI in models for rates of hospitalisation for both outcomes and one participant had missing BMI in models for case fatality rates for ischaemic heart disease. The area of each square is inversely proportional to the variance. GLM=generalised linear regression models. UEBMI=urban employee basic medical insurance. URBMI=urban resident basic medical insurance. NRCMS=new rural cooperative medical scheme. BMI=body-mass index.

In 2009, the rates of hospitalisation were higher in urban than in rural areas for stroke (13·5 *vs* 8·7 admissions per 1000 person-years) and for ischaemic heart disease (13·0 *vs* 6·3; figure 2). The absolute differences in hospitalisation rates between individuals living in urban and rural areas in 2009–16 only decreased for ischaemic heart disease (trend: p=0·013; appendix 2 p 11). The fully adjusted annual increases in hospitalisation rates were higher for individuals living in rural than in urban areas for stroke (4·5% *vs* 3·3%) and ischaemic heart disease (8·4% *vs* 3·6%; figure 3). Overall, the fully adjusted hospitalisation rates for stroke and ischaemic heart disease were similar by levels of education. However, individuals with no formal education had the greatest annual increase in hospitalisation rates for both stroke (7·1%) and ischaemic heart disease (8·2%). Similarly, individuals in the lowest income group had the highest annual increase in fully adjusted hospitalisation rates for stroke (7·0%) and ischaemic heart disease (9·8%). Individuals enrolled in UEBMI had higher rates of hospitalisation for stroke and ischaemic heart disease than those enrolled in URBMI or NRCMS (stroke: 12·7 *vs* 10·3 per 1000 person-years in 2009; ischaemic heart disease: 11·3 *vs* 8·1 per 1000 person-years in 2009; figure 2). The differences and slope index of inequality in hospitalisation rates between UEBMI and URBMI or NRCMS enrollees decreased over time for stroke (trend: differences p=0·039; slope p=0·014) and ischaemic heart disease (both p<0·0001; appendix 2 p 11). The annual increases in rates were higher for individuals enrolled in URBMI or NRCMS than in UEBMI (stroke: 4·2% *vs* 2·5%; ischaemic heart disease: 7·4% *vs* 3·6%; figure 3).

**Figure 4:**
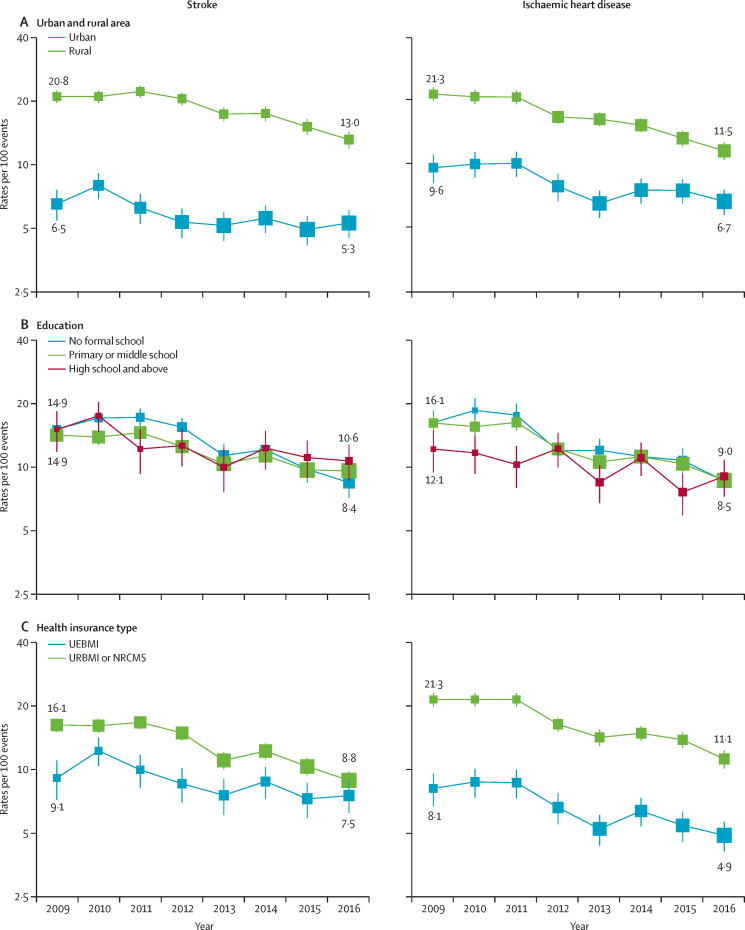
Annual 28-day case fatality rates for stroke and ischaemic heart disease, by urban and rural area, education, and health insurance type Generalised linear regression models with binomial distribution and logit link function were used. Fully adjusted models included adjustments for demographic, socioeconomic, lifestyle, and morbidity factors, as well as region and case mix. In analyses by health insurance type, uninsured participants were excluded because of the small number of cases. Rates by education and health insurance type were standardised for the overall CKB participant population in 2009 and rates by urban and rural area were standardised separately for the CKB participant population living in urban or rural areas, in 2009. One participant had missing data for body-mass index in models for ischaemic heart disease. Rates per 100 events are shown for the years 2009 and 2016. The area of each square is inversely proportional to the variance. 95% CIs are shown. UEBMI=urban employee basic medical insurance. URBMI=urban resident basic medical insurance. NRCMS=new rural cooperative medical scheme.

The fully adjusted 28-day case fatality rates for stroke decreased over time, from 14·4 to 9·3 deaths per 100 events (by 12·0% per year, 95% CI 10·3–13·7), and for ischaemic heart disease from 15·4 to 8·7 deaths per 100 events (by 14·5% per year, 12·6–16·3; appendix 2 p 17). The annual decreases in case fatality rates for stroke and ischaemic heart disease did not vary significantly between 2009–12 and 2013–16 (appendix 2 p 10). Case fatality rates for stroke and ischaemic heart disease were higher for individuals living in rural than in urban areas from 2009–16 (stroke: 13·0 *vs* 5·3 deaths per 100 events, ischaemic heart disease: 11·5 *vs* 6·7 in 2016; figure 4). However, the annual decrease in case fatality rates was around two-times higher for rural residents (stroke: 11·9% *vs* 6·4%; ischaemic heart disease: 16·7% *vs* 8·3%; figure 3). During the study period, case fatality rates decreased to a greater extent in individuals with lower levels of education or income (figure 3; appendix 2 p 18). Likewise, while 28-day case fatality rates for URBMI or NRCMS enrollees were higher than for UEBMI enrollees (stroke: 16·1 *vs* 9·1 deaths per 100 events; ischaemic heart disease: 21·3 *vs* 8·1 in 2009; figure 4), the annual decrease in case fatality rates was higher in URBMI or NRCMS enrollees (stroke: 14·2% *vs* 7·8%, ischaemic heart disease: 17·2% *vs* 11·2%; figure 3). Both the absolute differences and slope index of inequality in case fatality rates for stroke and ischaemic heart disease also decreased over time between individuals living in rural versus urban areas, those with the lowest versus highest levels of education, and those enrolled in URBMI or NRCMS versus UEBMI (all trends p<0·05; appendix 2 p 12).

**Figure 5:**
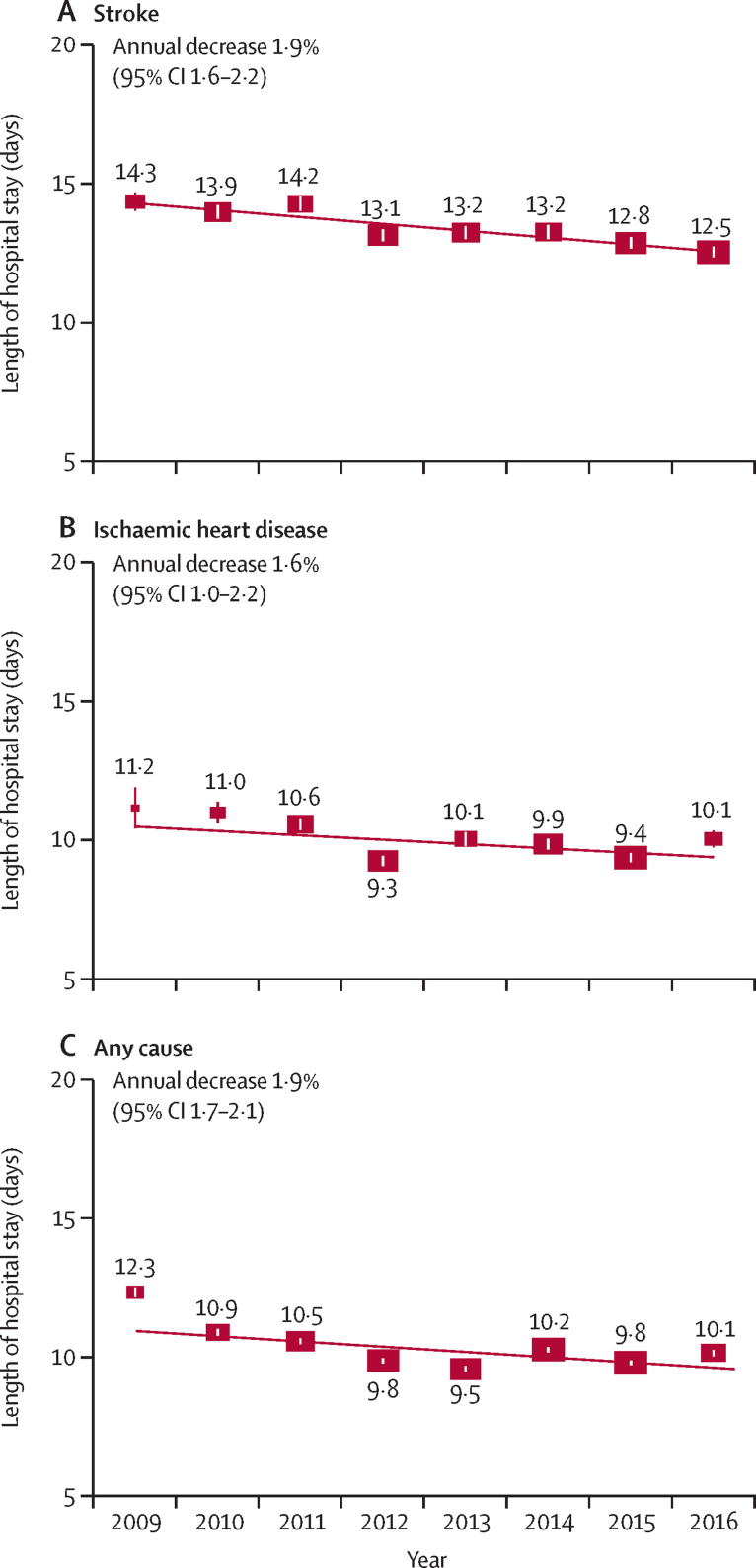
Mean length of hospital stay for stroke, ischaemic heart disease, and any cause, by year Generalised linear regression models with gamma distribution and log link function were used. Fully adjusted models included adjustments for demographic, socioeconomic, lifestyle, and morbidity factors, as well as region and case mix. Mean length of stay was standardised for the overall China Kadoorie Biobank participant population in 2009. Two participants had missing data for body-mass index in models for any cause. Numbers above the squares are mean length of stay in days. 95% CIs are shown. The area of each square is inversely proportional to the variance.

Between 2009 and 2016, the annual mean length of stay decreased by 1·9% per year for stroke (from 14·3 days in 2009 to 12·5 in 2016), 1·6% for ischaemic heart disease (from 11·2 days in 2009 to 10·1 in 2016), and 1·9% for any cause (from 12·3 days in 2009 to 10·1 in 2016; figure 5). Urban residents had a longer mean length of stay for stroke and ischaemic heart disease than rural residents (stroke: 13·0 *vs* 12·0 days; ischaemic heart disease: 10·8 *vs* 8·8, in 2016), and likewise for UEBMI versus URBMI or NRCMS enrollees (appendix 2 p 19). The annual decrease in mean length of stay was greater for individuals with the highest levels of education or income for stroke, ischaemic heart disease, and any cause for individuals living in urban areas and those enrolled in UEBMI for stroke and ischaemic heart disease (figure 3; appendix 2 p 20). The absolute differences in mean length of stay for stroke and ischaemic heart disease between the highest and lowest category and slope index of inequality decreased over time for most socioeconomic characteristics (appendix 2 p 13).

In sensitivity analyses, the fully adjusted probabilities of first-ever hospitalisation for stroke increased by 3·8% (95% CI 3·3–4·3) per year and by 5·8% (5·2–6·4) per year for ischaemic heart disease (appendix 2 p 21), which was similar to the increase for all admissions. Likewise, the mean length of stay of first-ever hospitalisation decreased by 2·4% (95% CI 2·0–2·9) per year for stroke and by 3·9% (3·0–4·9) for ischaemic heart disease. The annual decrease for first-ever hospitalisation was similar to that for all admissions for stroke, but was greater for ischaemic heart disease. Annual differences in the rates of hospital admissions and case fatalities between urban and rural areas were smaller when standardised across the entire study population, but without adjustment for region (appendix 2 p 14).

The annual increase in hospitalisation rates for stroke was mainly due to increases in admissions for ischaemic rather than haemorrhagic stroke (4·9%, 95% CI 3·5 to 4·4 *vs* 1·1%, −0·2 to 2·3; appendix 2 p 22), while the annual increase in rates of hospitalisation for ischaemic heart disease was due to increases in both acute myocardial infarction (6·4%, 95% CI 4·6 to 8·3) and other ischaemic heart disease (5·3%, 4·8 to 5·8). Fully adjusted annual rates of hospitalisation, case fatality rates, and mean length of stay for stroke, ischaemic heart disease, and any cause were similar across age groups, and annual percentage changes by age group were also broadly similar to the overall annual percentage change (appendix 2 pp 15, 23).

## Discussion

We studied around 0·5 million Chinese adults who were followed up for an 8-year period from 2009 to 2016. We found that, after controlling for demographic, socioeconomic, lifestyle, and morbidity factors, hospital admissions for stroke and ischaemic heart disease increased by approximately 4–5% per year. Moreover, although hospital use was higher among individuals living in urban areas and those enrolled in UEBMI, annual increases in hospitalisation rates for stroke and ischaemic heart disease were greater among individuals living in rural areas, those with lower levels of education or income, and those enrolled in URBMI or NRCMS than UEBMI. In addition, the study showed that case fatality rates for stroke and ischaemic heart disease decreased over the period. While case fatality rates were higher in lower than higher socioeconomic groups, annual reductions in case fatality rates were greater in the lower socioeconomic groups. Over the same period, the mean length of stay decreased by around 2% per year for stroke, ischaemic heart disease, and any cause. The mean length of stay was longer for individuals living in urban areas and enrolled in UEBMI, and the annual decrease in mean length of stay was higher in these groups and in individuals with higher education or income levels. These trends indicate modest improvements in use, quality, and efficiency of hospital care for stroke, ischaemic heart disease, and any cause in China.

National statistics and previous studies in China also reported increased rates of hospital admissions for any cause and cardiovascular disease over the calendar period of the present study.^5^, ^10^, ^14^, ^16^, ^22^ However, age, sex, and region-adjusted admission rates for any cause in the CKB study were higher (23% for CKB *vs* 17% for national data in 2016), which probably reflects the older population studied in the CKB study.^23^ Annual increases in hospitalisation rates for stroke, ischaemic heart disease, and any cause were reduced by around half after adjustment for socioeconomic, lifestyle, and morbidity factors. The observed reduction was consistent with reports that a large proportion of the increasing cardiovascular disease burden in China is likely to reflect poor control of established cardiovascular disease risk factors (eg, smoking, systolic blood pressure, LDL cholesterol, blood glucose, body-mass index, and physical activity).^24^

Apart from established cardiovascular disease risk factors and morbidities, health system factors could also play an important role in the increasing trends in hospital use.^5^ Although the present trends cannot attribute causality between health-care reforms and changes in hospital use and outcomes between different socioeconomic groups, the present findings could inform future public health policy to reduce inequalities in health-care delivery in China. These results suggest greater increases in hospital use during the first cycle of the reforms (2009–12), coinciding with a greater expansion of health insurance coverage.^25^ During the reform period, inpatient reimbursement rates and ceilings (maximum level of patient reimbursement) for NRCMS and URBMI increased rapidly and deductibles decreased, which might partly account for the higher annual increases in hospital use for enrollees of such schemes compared with UEBMI enrollees.^5^ In 2012, China launched a critical illness insurance, which covers patients enrolled in NRCMS or URBMI with diseases whose annual health-care costs exceed the annual mean disposable income per capita in the local area.^26^ Individuals who have critical illnesses, including stroke and ischaemic heart disease subtypes, receive extra reimbursements for treatment costs,^5^ which increases affordable access to hospital care. As part of the reforms, local civil affairs bureaus designed medical assistance programmes to cover any remaining co-payments for priority diseases and individual contributions for low-income households.^6^ Our study showed that the most disadvantaged groups had the highest annual increases in rates of hospitalisation and greatest reductions in case fatality rates. However, inequalities in hospitalisation and case fatality rates between different socioeconomic groups have persisted between 2009 and 2016, possibly reflecting further differences in health-care seeking and affordability.

The increasing rates of hospital use, although at a lower rate for urban residents and higher socioeconomic groups, might reflect the financial incentives of hospitals to promote greater use of hospital services and underdeveloped and underused primary health care in China.^25^, ^27^, ^28^ Patients' distrust of primary health-care services combined with the design of health insurance packages, which continue to provide low levels of coverage for outpatient care compared with inpatient care, are key contributors.^5^, ^6^ Moreover, between 2009 and 2016, the number of hospitals in China increased by 44% nationally, and the number of beds in medical institutions increased by 42% in urban areas and by 50% in rural areas, which might also have contributed to the increased hospital use.^23^

The decreasing trends in case fatality rates among all socioeconomic groups might partly reflect improvements in quality of care and more effective hospital treatment.^29^, ^30^ The findings in the present study are consistent with the substantial reductions in case fatality rates for stroke and ischaemic heart disease reported in western countries.^30^, ^31^, ^32^, ^33^, ^34^ Case fatality rates for stroke in the present study were lower than those previously reported by other studies in China (<5–50%).^27^, ^35^ Lower rates in the present study might reflect differences in disease severity, types of stroke, quality of care, or lower thresholds for diagnosis over time.^27^, ^35^

Decreasing annual trends in mean length of stay for stroke, ischaemic heart disease, and any cause probably reflect the government introduction of a target mean length of stay as a performance indicator to reduce health-care expenditure, and other efforts by health insurance agencies to reduce costs.^36^ China has also implemented standard clinical pathways for major cardiovascular disease outcomes (eg, acute ischaemic stroke and myocardial infarction) and established an official monitoring system for these pathways in 2009.^29^ Such reforms appear to have contributed to reductions in mean length of stay, and improvements in survival after stroke and ischaemic heart disease.^37^ The greater reduction in mean length of stay for higher socioeconomic groups might reflect the greater reductions in mean length of stay in large urban hospitals, mostly used by these groups.^38^ However, mean length of stay in China is still greater than that in Organisation for Economic Co-operation and Development member countries,^34^ with the typically conservative behaviour of Chinese doctors seeking to comply with patient wishes; the adverse incentives generated by the use of fee-for-service reimbursement;^12^, ^36^ and the limited availability of rehabilitation services for post-acute care identified as contributors.^29^

The main strengths of the present study include a long duration of follow-up, which covers the two major waves of the reforms (2009–12 and 2013–16);^25^ the diversity in regions and hospitals included; and the extensive data collected for individual participants, which enabled adjustment for a wide range of confounders. However, the study also had several limitations. First, the CKB was designed to include diverse areas, socioeconomic groups, and risk factors in China rather than to be representative of the Chinese population, so results should be interpreted with caution. However, the diverse socioeconomic groups studied suggest that the findings are likely to be generalisable to the full range of such exposures in the population. Second, despite our ability to adjust for participant's characteristics at entry and their age and morbidities during follow-up, the study was constrained by having a longitudinal fixed cohort study design. In the CKB study, periodic resurveys only included a random subset of participants (around 5%), so some of the covariates included in the analyses could not be updated during follow-up. Third, data for participants' health insurance type were only available from 2012 onwards and, therefore, an assumption had to be made that the participants' health insurance type in 2009–11 was the same as that recorded in 2012. However, data from resurveys of CKB participants indicated that 97% of participants reported being insured in both 2008 and 2014, and the proportion of participants enrolled in the same scheme between 2012 and 2016 remained stable. Fourth, the data available in the present study did not allow for a more nuanced measure of hospital care access with hospital use capturing exclusively health demand presenting for hospital care. Fifth, interpreting fully adjusted trends in hospitalisation rates as health system effects relies on the assumption that adjustments for demographic (eg, age and sex), socioeconomic, lifestyle, and morbidity factors were successful. Fully adjusted trends also need to be interpreted in light of other possible supply and demand factors, including details of health insurance coverage and health-care costs, hospital-level characteristics, distance between household and hospitals, outpatient care provision and use, physician-related factors, and changes in admission thresholds or use of diagnostic tests for cardiovascular disease.^27^ Sixth, as participants in each CKB region were either all urban or all rural residents, absolute differences in hospital use and disease outcomes between urban and rural areas, although subject to regional variation, could not be adjusted for differences across all regions. Overall, the associations reported in this prospective cohort study cannot be causally attributed to the health-care reforms as a different study design would be required to address causality. Longer duration of follow-up might also be needed to reliably assess the longer-term effects of the health-care reforms in China.

In conclusion, this large prospective study examined changes in hospital care use and outcomes for individuals with stroke and ischaemic heart disease between 2009 and 2016, both overall and across different socioeconomic groups. The robust findings of increasing hospital admission rates and decreasing case fatality rates, particularly in rural areas and for individuals with lower levels of education or income, and enrolled in the urban or rural resident health insurance scheme, reflect improvements in use and quality of care in 2009–16. The continuous reductions in mean length of stay also indicate improvements in hospital efficiency. Nevertheless, there is substantial scope to achieve further reductions in socioeconomic inequalities, by altering health insurance benefits, strengthening primary care, and implementing cost-effective interventions to decrease the cardiovascular disease burden and control escalating hospital care costs.

## Supplementary Materials

Chinese translation of the abstract
