# Supplementary material for: Socioeconomic differences in health-care use and outcomes for stroke and ischaemic heart disease in China during 2009–16: a prospective cohort study of 0·5 million adults
Source: Lancet Glob Health. 2020 Mar 18;8(4):e591–602. doi: 10.1016/S2214-109X(20)30078-4 (PMC7090927; doi:10.1016/S2214-109X(20)30078-4)
Supplement: Chinese translation of the abstract [file mmc1.pdf]

THE LANCET
Global Health

Supplementary appendix 1

This translation in Chinese was submitted by the authors and we reproduce it as supplied. It has not been peer reviewed. *The Lancet*'s editorial processes have only been applied to the original in English, which should serve as reference for this manuscript.

此简体中文译文由作者提交，我方按照提供的版本刊登。此译文并未经过同行审阅。医学期刊《柳叶刀》的编辑流程仅适用于英文原稿，英文原稿应作为此手稿的参考。

Supplement to: Levy M, Chen Y, Clarke R, et al. Socioeconomic differences in health-care use and outcomes for stroke and ischaemic heart disease in China during 2009–16: a prospective cohort study of 0·5 million adults. *Lancet Glob Health* 2020; **8:** e591–602.

背景

中国于 2009 年启动了重大医疗改革，旨在于 2020 年之前实现全民医保覆盖。但是，对于医改所带来的不同社会经济地位群体在医疗资源的利用和健康状况的改善等方面的变化趋势，我们还知之甚少。

研究方法

自 2004 至 2008 年，中国慢性病前瞻性研究调查了来自全国 10 个（5 个城市，5 个农村）地区的 50 万成年人（基线年龄 30-79 岁）。于 2009 至 2016 年的 7 年间，一共 794 824 例（人次）住院病例，其中包括中风 74 313 例和缺血性心脏病 69 446 例。我们用广义线性模型来估计中风、缺血性心脏病和年度总住院率、28 天病死率和平均住院时间的趋势。

结果

在对人口学、社会经济地位、生活方式和主要并发疾病等因素进行校正之后，中风，缺血性心脏病和总住院率在 2009 年至 2016 年之间分别每年增长 3·6%,5·4%和 4·2%。较高社会经济地位群体的住院率较高，但较低社会经济地位群体有较高的年增长比例。对于中风而言，农村地区的年增比例[95%可信区间]高于城市地区（4·5%[3·9,5·1]对 3·3%[2·8,3·9]），教育程度较低群体明显高于高等教育人群（7·1%[6·2,8·1]对 2·9%[2·0,3·7]），收入较低群体显著高于收入较高群体（7·0%[6·3,7·8] vs 2·0%[1·1,3·0]）。对于缺血性心脏病，以上各人群相应的年增长比例分别为 8·4%[7·6,9·2]对 3·6%[3·0,4·2]、8·2%[6·9,9·4]对 3·3%[2·4,4·2]、以及 9·8%[8·8,10·3]对 3·2%[2·1,4·4]。农村和城市居民医保用户的住院率年增长比例高于城市职工医保用户（中风: 4·2%[3·6,4·8] vs 2·5%[3·6,4·8]; 缺血性心脏病: 7·4%[6·6,8·1]和 3·6%[2·9,4·2]。较低社会经济地位群体中风和缺血性心脏病的病死率更高，但与较高社会经济地位群体相比，病死率的降低幅度也更大。相反，中风、缺血性心脏病和总住院事件的平均住院时间每年下降约 2%，其中中风和缺血性心脏病的下降幅度在社会经济地位较高的人群中更大。

解读

2009 年至 2016 年间，虽然社会经济地位较高的群体住院率较高，但社会经济地位群体的住院率上升幅度更大，他们的中风和缺血性心脏病病死率下降幅度也更大。但是，我们仍然需要采取进一步的措施来减少由于社会经济地位的不同所造成的在医保的使用和疾病转归方面的差异。
