# Supplementary material for: Socioeconomic differences in health-care use and outcomes for stroke and ischaemic heart disease in China during 2009–16: a prospective cohort study of 0·5 million adults
Source: Lancet Glob Health. 2020 Mar 18;8(4):e591–602. doi: 10.1016/S2214-109X(20)30078-4 (PMC7090927; doi:10.1016/S2214-109X(20)30078-4)
Supplement: Supplementary appendix [file mmc2.pdf]

THE LANCET
Global Health

Supplementary appendix 2

This appendix formed part of the original submission and has been peer reviewed. We post it as supplied by the authors.

Supplement to: Levy M, Chen Y, Clarke R, et al. Socioeconomic differences in health-care use and outcomes for stroke and ischaemic heart disease in China during 2009–16: a prospective cohort study of 0·5 million adults. *Lancet Glob Health* 2020; **8:** e591–602.

Supplementary Appendix 2

Supplement to: Levy M, Chen Y, Clarke R, et al. Socioeconomic differences in health-care use and outcomes for stroke and ischaemic heart disease in China during 2009-16: a prospective cohort study of 0·5 million adults

Table of Contents

Page		
2	Table of contents	
4	Members of the China Kadoorie Biobank Collaborative Group	
5	Supplementary Methods	
9	webtable 1:	Number of participants by socioeconomic category, in each CKB region
10	webtable 2:	Fully adjusted annual percentage change in rates of hospitalisation, mean length of hospital stay and 28-day case fatality rates (95% CI) for stroke, ischaemic heart disease and any cause during the two cycles of healthcare reforms in China in the period 2009-2016
11	webtable 3:	Absolute differences in fully adjusted rates of hospitalisation between highest and lowest category of each socioeconomic characteristic and slope index of inequality (SII) (per 1000 person-years), by calendar year
12	webtable 4:	Absolute differences in fully adjusted 28-day case fatality rates between lowest and highest category of each socioeconomic characteristic and slope index of inequality (SII) (per 100 events), by calendar year
13	webtable 5:	Absolute differences in fully adjusted mean length of stay between highest and lowest category of each socioeconomic characteristic and slope index of inequality (SII) (in days), by calendar year
14	webtable 6:	Absolute annual differences in adjusted rates of hospitalisation, 28-day case fatality rates, mean length of hospital stay and slope index of inequality (SII) between rural and urban participants, without region adjustment
15	webtable 7:	Annual percentage change in fully adjusted rates of hospitalisation, 28-day case fatality and mean length of stay for stroke and ischaemic heart disease, by age group
16	webfigure 1:	Annual unadjusted and fully adjusted rates of hospitalisation for stroke, ischaemic heart disease and any cause
17	webfigure 2:	Fully adjusted 28-day case fatality rates for stroke and ischaemic heart disease, by calendar year

18 webfigure 3: Fully adjusted annual rates of hospitalisation, 28-day case fatality and mean length of stay for stroke and ischaemic heart disease, by income

19 webfigure 4: Fully adjusted annual mean length of hospital stay for stroke and ischaemic heart disease, by urban and rural area, education and health insurance type

20 webfigure 5: Fully adjusted annual percentage change in rates of hospitalisation and mean length of hospital stay for any cause, by urban and rural area, education, income and health insurance type

21 webfigure 6: Fully adjusted rates of hospitalisation and mean length of hospital stay for first-ever hospital admission for stroke and ischaemic heart disease

22 webfigure 7: Fully adjusted rates of hospitalisation and mean length of hospital stay, by type of stroke and ischaemic heart disease

23 webfigure 8: Fully adjusted rates of hospitalisation, 28-day case fatality rates and mean length of hospital stay for stroke and ischemic heart disease, by age group

Members of the China Kadoorie Biobank Collaborative Group

International Steering Committee: Junshi Chen, Zhengming Chen (PI), Robert Clarke, Rory Collins, Yu Guo, Liming Li (PI), Jun Lv, Richard Peto, and Robin Walters.
International Co-ordinating Centre, Oxford: Daniel Avery, Derrick Bennett, Ruth Boxall, Fiona Bragg, Yumei Chang, Yiping Chen, Zhengming Chen, Robert Clarke, Huaidong Du, Simon Gilbert, Alex Hacker, Michael Holmes, Christiana Kartsonaki, Rene Kerosi, Garry Lancaster, Kuang Lin, John McDonnell, Iona Millwood, Qunhua Nie, Paul Ryder, Sam Sansome, Dan Schmidt, Rajani Sohoni, Iain Turnbull, Robin Walters, Jenny Wang, Lin Wang, Neil Wright, Ling Yang, and Xiaoming Yang.

National Co-ordinating Centre, Beijing: Zheng Bian, Yu Guo, Xiao Han, Can Hou, Biao Jing, Chao Liu, Jun Lv, Pei Pei, and Canqing Yu.

Regional Co-ordinating Centres: Qingdao **Qingdao** CDC: Zengchang Pang, Ruqin Gao, Shanpeng Li, Shaojie Wang, Yongmei Liu, Ranran Du, Yajing Zang, Liang Cheng, Xiaocao Tian, Hua Zhang, Yaoming Zhai, Feng Ning, Xiaohui Sun, Feifei Li. Licang CDC: Silu Lv, Junzheng Wang, Wei Hou. **Heilongjiang** Provincial CDC: Mingyuan Zeng, Ge Jiang, Xue Zhou. Nangang CDC: Liqiu Yang, Hui He, Bo Yu, Yanjie Li, Qinai Xu, Quan Kang, Ziyan Guo. **Hainan** Provincial CDC: Dan Wang, Ximin Hu, Hongmei Wang, Jinyan Chen, Yan Fu, Zhenwang Fu, Xiaohuan Wang. Meilan CDC: Min Weng, Zhendong Guo, Shukuan Wu, Yilei Li, Huimei Li, Zhifang Fu. **Jiangsu** Provincial CDC: Ming Wu, Yonglin Zhou, Jinyi Zhou, Ran Tao, Jie Yang, Jian Su. Suzhou CDC: Fang Liu, Jun Zhang, Yihe Hu, Yan Lu, Liangcai Ma, Aiyu Tang, Shuo Zhang, Jianrong Jin, Jingchao Liu. **Guangxi** Provincial CDC: Zhenzhu Tang, Naying Chen, Ying Huang. Liuzhou CDC: Mingqiang Li, Jinhuai Meng, Rong Pan, Qilian Jiang, Jian Lan, Yun Liu, Liuping Wei, Liyuan Zhou, Ningyu Chen, Ping Wang, Fanwen Meng, Yulu Qin, Sisi Wang. **Sichuan** Provincial CDC: Xianping Wu, Ningmei Zhang, Xiaofang Chen, Weiwei Zhou. Pengzhou CDC: Guojin Luo, Jianguo Li, Xiaofang Chen, Xunfu Zhong, Jiaqiu Liu, Qiang Sun. **Gansu** Provincial CDC: Pengfei Ge, Xiaolan Ren, Caixia Dong. Maiji CDC: Hui Zhang, Enke Mao, Xiaoping Wang, Tao Wang, Xi Zhang. **Henan** Provincial CDC: Ding Zhang, Gang Zhou, Shixian Feng, Liang Chang, Lei Fan. Huixian CDC: Yulian Gao, Tianyou He, Huarong Sun, Pan He, Chen Hu, Xukui Zhang, Huifang Wu, Pan He. **Zhejiang** Provincial CDC: Min Yu, Ruying Hu, Hao Wang. Tongxiang CDC: Yijian Qian, Chunmei Wang, Kaixu Xie, Lingli Chen, Yidan Zhang, Dongxia Pan, Qijun Gu. **Hunan** Provincial CDC: Yuelong Huang, Biyun Chen, Li Yin, Huilin Liu, Zhongxi Fu, Qiaohua Xu. Liuyang CDC: Xin Xu, Hao Zhang, Huajun Long, Xianzhi Li, Libo Zhang, and Zhe Qiu.

Supplementary Methods

Baseline questionnaires

Data on demographic, socioeconomic characteristics, lifestyle, and medical history were collected using an interviewer-administered, laptop-based electronic questionnaire (with logic checks to minimise missing values, errors and inconsistencies) administered to study participants. Extensive training was provided for field survey staff, including instruction on data collection using the electronic questionnaire, recording physical measurements using standard protocols and on use and maintenance of equipment. Importantly, serial resurveys in 5% of the study population indicated a high level of agreement between replicate measurements for questionnaire data and clinical measurements.

Follow-up data

Data on hospital admissions were obtained from both HI records and disease registers to include both insured and uninsured individuals. Disease registers were prospectively set up by CKB Regional Coordinating Centres to record all hospital admissions for stroke, IHD, cancer and diabetes. Based on the initial linkage established in study areas, personal data have been matched to the HI system for almost all participants using the unique national identity number (ID). Linkage to HI records in all CKB participants was completed for all centres from 2008/2009 onwards. The HI and vital status of participants unlinked to the HI system were checked annually by local public health specialists. Between 2009 and 2016, the proportions of admissions available from HI records for stroke, IHD and any cause were on average 83%, 92% and 97%, respectively.

Cause-specific mortality for each participant was monitored through China's Disease Surveillance Points and Information system (DSP) of the Chinese Centre for Diseases Control and Prevention (CDC), which covers all study areas. Annual checks with local residential records, HI records, and active confirmation of survival were conducted by local residential administrators to minimize any under-reporting of deaths and identify participants who have moved permanently out of the study areas. The DSP provided reliable and complete registration for causes of death in almost all adults. For any additional deaths not identified through routine procedures (<5%), the causes were sought by reviewing hospital records or by conducting a verbal autopsy using a validated instrument (Chen Z et al., 2011). Between entry into the CKB study and 31 December 2016, only 4751 (0.9%) individuals were lost to follow-up (251 individuals in 2005-2008). Participants who permanently moved out of the region where they were recruited were considered to be lost to follow-up. These participants did not contribute to the analyses for the years after they were lost to follow-up and statistical models were adjusted for the proportion of time that the participants were observed in the study.

Imputation methods for missing length of stay

Hospital admissions data were obtained from both HI records and disease registers. Disease registers only included admission dates for all events, but did not include discharge dates. For all admissions obtained from disease registers, the length of stay could not be calculated and was denoted as being missing (stroke: 12 586 [16.9%], IHD: 5535 [8.0%], any cause: 30 232 [3.8%]).

We used multiple imputation with Poisson regression adjusted with the same covariates (eg, calendar years, demographic, socioeconomic, lifestyle, and morbidity factors, region and type of stroke and IHD) as included in fully adjusted model when estimating mean LOS overall. The number of imputations (*m*) was chosen based on: m≥100*Fraction of Missing Information (White et al., 2011). We used 20, 10 and 5 imputations in models for stroke, IHD and any cause, respectively.

Selection of individual Generalised Linear Models (GLM)

Using minimally adjusted models, the Modified Park Test was used to test which model had the best fit and the Pearson Correlation Test, Pregibon Link Test and Modified Hosmer and Lemeshow Tests were used to test the link.

For rates of hospitalisation, the Poisson Family was preferred over Gamma, Gaussian NLLS and Inverse Gaussian or Wald models, and the Log link was preferred over the Identity and Square root link. We then compared models using Poisson and Negative Binomial distributions, and selected the Negative Binomial distribution as the Likelihood Ratio Test indicated a significant over-dispersion parameter (i.e. over dispersion of 0 count; $p<0.001$) for this model.

Values of mean LOS were positive and their distribution was highly skewed. For mean LOS, the Modified Park Test indicated that the Gamma family was preferred (any cause: coefficient 2.03) and results for tests of GLM Log link were all non-significant (any cause: Pearson Correlation Test: 0.08, Pregibon Link Test: 0.13, Modified Hosmer and Lemeshow: 0.41).

Imputation methods used for missing health insurance scheme before 2012

Participants were linked to individual HI schemes annually starting from 2012, using the participants' unique national ID number. Information was also provided on any uninsured participants each year.

Given the follow-up period was limited to the interval between 2009 and 2016, data for participants' HI schemes for the period 2009-2011 were imputed using the earliest participant's HI schemes available between 2012 and 2016. In China, individuals are enrolled in a particular HI scheme depending on their employment status. The proportion of CKB participants insured in the same scheme remained stable between 2012 and 2016: NRCMS/URBMI (56.1-56.5%), UEBMI (37.5-38.9%), unknown: (0.9-0.6%), uninsured (5.5-3.9%). Furthermore, at entry into the study (2004-2008), 82% of participants self-reported being insured. This proportion increased to 97% and 98% in the 5% sample of individuals who participated in the 1st resurvey

(2008) and the 2nd resurvey (2013-2014), respectively. Thus, it was unlikely for participants to be uninsured. Uninsured participants were excluded from analyses by HI type, due to small number of cases.

In the imputation for HI schemes, an assumption was made that middle-aged and older individuals in China such as participants in CKB (ie, mean age 54.7 years in 2009) were unlikely to change employment and, hence, their HI scheme. The imputation was performed as outlined below:

(1) The "unknown" HI scheme was used to code participants that were insured, but whose HI type could not be identified. To reduce the proportion of unknown HI types in 2012-2016, we first imputed the unknown scheme using the HI scheme that participants were enrolled in during the previous year (if not uninsured or unknown), and then using the HI scheme they were enrolled in during the following year (if not uninsured or unknown).
(2) Between 2009 and 2011, all participants were assumed to be enrolled in the same HI scheme as in 2012.
(3) For participants uninsured in 2012 (5.5%), we checked whether they had any admission reported in HI records in 2009-2011. If they had an admission, we coded them as having unknown HI type for that year.
(4) Participants censored before 2012 (0.6%) were assumed to be insured and their HI type was replaced by a "Missing" scheme.
(5) Participants with missing and unknown HI scheme in 2009-2016 (1.4%), had their HI scheme replaced by one of the three main schemes (UEBMI, URBMI, NRCMS) based on their self-reported occupation at entry into the study:
    a) For participants that reported being agriculture or related workers, we replaced their missing/unknown scheme with NRCMS.
    b) For participants living in rural areas, we replaced their missing/unknown scheme by NRCMS, as the majority of individuals living in rural areas were enrolled in this scheme in 2012-2016.
    c) For participants living in urban areas and in formal employment (factory worker, sales and services, administration/manager, professional/technical), their missing scheme was replaced by UEBMI.
    d) For participants living in urban areas and in informal employment (housewife/househusband, unemployed, other or non-stated), we replaced their missing/unknown scheme by URBMI.
    e) For retired individuals living in urban areas, if they were male and aged ≥60 or female and aged ≥55 (official retirement ages), their missing/unknown scheme was replaced by UEBMI, as the majority of retired individuals in urban areas were enrolled in UEBMI in 2012-2016.
    f) Remaining participants with an unknown/missing information about scheme (0.03% in 2009-2016) were combined with the uninsured category.

Description of different types of health insurance schemes in China

(i) UEBMI (Urban Employee Basic Medical Insurance): compulsory scheme for urban employees. This scheme is funded by both employer (8-10%) and employee (2%) contributions. Retired individuals who were previously employed and covered by UEBMI remain enrolled in UEBMI.
(ii) URBMI (Urban Resident Basic Medical Insurance): voluntary scheme for children, students, urban residents without formal employment and elderly without previous employment. This scheme is mainly funded by government subsidies (~70% of the total funds).
(iii) NRCMS (New Rural Cooperative Medical Scheme): voluntary scheme for rural residents. This scheme is mainly funded by government subsidies (~70% of the total funds).

Summary statistics of admissions for stroke, IHD or any cause

Between 2009 and 2016, 86.4% of participants had no hospital admission, 9.9% had 1 admission, 2.3% had 2 admissions and 1.4% had more than 2 admissions for any cause. For stroke, 98.4% of participants had no admission, 1.4% had 1 admission, 0.2% had 2 admissions and 0.04% had more than 2 admissions. For IHD, 98.4% of participants had no admission, 1.4% had 1 admission, 0.2% had 2 admissions and 0.04% had more than 2 admissions.

webtable 1: Number of participants by socioeconomic category, in each CKB region in 2009

		Education, n (%)			Household income (¥/year), n (%)				Health insurance type in 2012, n (%)		
		No formal school	Primary/ middle school	High school and above	<10,000	10,000-19,999	20,000-34,999	≥35,000	URBMI or NRCMS	UEBMI	Uninsured/ Others
**Urban**	**Qingdao n=35 202**	2025 (5·8)	20 614 (58·6)	12 563 (35·7)	2748 (7·8)	11 258 (32·0)	15 302 (43·5)	5 894 (16·7)	689 (2·0)	33 204 (95·7)	793 (2·3)
	**Harbin n=56 763**	2305 (4·1)	23 056 (40·6)	31 402 (55·3)	7129 (12·6)	19 028 (33·5)	17 800 (31·4)	12 806 (22·6)	3646 (6·6)	47 556 (85·9)	4189 (7·6)
	**Haikou n=29 568**	4347 (14·7)	14 097 (47·7)	11 124 (37·6)	6504 (22·0)	9382 (31·7)	6464 (21·9)	7218 (24·4)	10 967 (37·5)	12 760 (43·6)	5531 (18·9)
	**Suzhou n=52 795**	15 699 (29·7)	31 925 (60·5)	5171 (9·8)	6267 (11·9)	7650 (14·5)	16 729 (31·7)	22 149 (42·0)	22 580 (43·4)	24 329 (46·7)	5160 (9·9)
	**Liuzhou n=49 765**	2058 (4·1)	27 374 (55·0)	20 333 (40·9)	7540 (15·2)	18 118 (36·4)	15 200 (30·5)	8907 (17·9)	6623 (13·6)	40 439 (83·0)	1648 (3·4)
	**Total n=281 902**	26 434 (11·8)	117 066 (52·2)	80 593 (36·0)	30 188 (13·5)	65 436 (29·2)	71 495 (31·9)	56 974 (25·4)	44 505 (20·2)	158 288 (71·9)	17 321 (7·9)
**Rural**	**Sichuan n=54 619**	8257 (15·1)	41 953 (76·8)	4409 (8·1)	34 002 (62·3)	15 665 (28·7)	3131 (5·7)	1821 (3·3)	39 477 (74·4)	11 599 (21·9)	1986 (3·7)
	**Gansu n=48 880**	22 274 (45·6)	22 140 (45·3)	4466 (9·1)	38 165 (78·1)	9623 (19·7)	977 (2·0)	115 (0·2)	44 815 (94·5)	1100 (2·3)	1524 (3·2)
	**Henan n=62 401**	8085 (13·0)	44 571 (71·4)	9745 (15·6)	25 612 (41·0)	27 600 (44·2)	7405 (11·9)	1784 (2·9)	56 151 (92·4)	1986 (3·3)	2606 (4·3)
	**Zhejiang n=57 027**	24 994 (43·8)	29 840 (52·3)	2193 (3·9)	3824 (6·7)	8061 (14·1)	23 580 (41·4)	21 562 (37·8)	43 747 (78·3)	10 592 (19·0)	1530 (2·7)
	**Hunan n=58 975**	2921 (5·0)	50 709 (86·0)	5354 (9·1)	9633 (16·3)	20 758 (35·2)	19 108 (32·4)	9476 (16·1)	52 272 (91·5)	2804 (4·9)	2060 (3·6)
	**Total n=281 902**	66 531 (23·6)	189 207 (67·1)	26 164 (9·3)	111 236 (39·5)	81 707 (29·0)	54 201 (19·2)	34 758 (12·3)	236 462 (86·2)	28 081 (10·2)	9706 (3·5)

webtable 2: Fully adjusted annual percentage change in rates of hospitalisation, mean length of hospital stay and 28-day case fatality rates (95% CI) for stroke, ischaemic heart disease and any cause during the two cycles of healthcare reforms in China in the period 2009-2016

		Stroke	Ischaemic heart disease	Any cause
**Rates of hospitalisation**	**1st cycle (2009-2012)**	5.7 (4.4, 7.0)	7.0 (5.4, 8.5)	5.0 (4.6, 5.5)
	**2nd cycle (2013-2016)**	0.5 (-0.4, 1.5)	2.6 (1.5, 3.6)	-0.7 (-1.1, -0.4)
**Mean length of hospital stay**	**1st cycle (2009-2012)**	-3.1 (-4.0, -2.3)	-6.5 (-7.9, -5.1)	-6.8 (-7.2, -6.3)
	**2nd cycle (2013-2016)**	-2.1 (-2.8, -1.5)	-0.5 (-1.5, 0.5)	0.8 (0.4, 1.2)
**28-day case fatality rates**	**1st cycle (2009-2012)**	-4.7 (-9.6, 0.6)	-13.0 (-17.8, -7.8)	
	**2nd cycle (2013-2016)**	-7.7 (-12.4, -2.8)	-10.8 (-15.4, -5.8)	

For rates of hospitalisation: Generalised Linear regression Models (GLM) with Negative Binomial distribution and log link function were used. For mean LOS: GLM with Gamma distribution and log link function were used. For 28-day case fatality rates, GLM with Binomial distribution and logit link function were used. Fully adjusted models included adjustments for demographic, socioeconomic, lifestyle, and morbidity factors, and region (and stroke or IHD type for mean LOS and case fatality rates). Exclusions as in Figure 3.

webtable 3: Absolute differences in fully adjusted rates of hospitalisation between highest and lowest category of each socioeconomic characteristic and slope index of inequality (SII) (per 1000 person-years), by calendar year

	Stroke					Ischaemic heart disease				
	Urban/Rural	Education		HI		Urban/Rural	Education		HI	
	Diff[1]	Diff[1]	SII[2]	Diff[1]	SII[2]	Diff[1]	Diff[1]	SII[2]	Diff[1]	SII[2]
**2009**	4.81 (0.34)	0.07 (0.50)	-0.04 (0.50)	2.43 (0.36)	4.78 (0.58)	6.66 (0.34)	-0.19 (0.53)	-0.07 (0.52)	3.15 (0.36)	6.20 (0.58)
**2010**	4.66 (0.34)	1.23 (0.50)	1.39 (0.51)	2.34 (0.36)	4.62 (0.58)	7.52 (0.34)	1.68 (0.54)	2.10 (0.53)	3.82 (0.37)	7.50 (0.60)
**2011**	6.82 (0.33)	0.61 (0.50)	0.80 (0.50)	4.04 (0.36)	7.93 (0.58)	9.05 (0.34)	2.64 (0.52)	3.54 (0.51)	5.65 (0.37)	11.12 (0.64)
**2012**	6.78 (0.34)	-1.46 (0.54)	-1.64 (0.53)	3.45 (0.38)	6.80 (0.61)	5.65 (0.34)	0.16 (0.57)	0.47 (0.56)	3.05 (0.41)	6.00 (0.68)
**2013**	7.74 (0.35)	-1.27 (0.53)	-1.57 (0.53)	3.33 (0.38)	6.56 (0.61)	7.13 (0.35)	-1.12 (0.53)	-1.56 (0.53)	2.75 (0.39)	5.42 (0.64)
**2014**	5.71 (0.32)	-1.86 (0.51)	-2.19 (0.50)	1.80 (0.35)	3.56 (0.56)	6.25 (0.34)	-2.66 (0.55)	-3.10 (0.54)	1.32 (0.38)	2.61 (0.62)
**2015**	6.41 (0.31)	-2.30 (0.49)	-2.53 (0.48)	2.16 (0.34)	4.28 (0.54)	7.88 (0.32)	0.37 (0.50)	0.72 (0.48)	3.21 (0.35)	6.32 (0.57)
**2016**	5.50 (0.32)	-3.06 (0.51)	-3.45 (0.50)	2.24 (0.35)	4.43 (0.56)	5.74 (0.33)	-3.33 (0.56)	-3.64 (0.53)	2.24 (0.39)	4.42 (0.63)
**p-value for trend**	0.0155	<0.0001	<0.0001	0.0392	0.0138	0.0113	<0.0001	<0.0001	<0.0001	<0.0001

[1]Diff: absolute difference in predicted rates between the highest and lowest group of each socioeconomic characteristic. [2]SII: absolute difference in predicted rates between the highest (ridit score of 1) and the lowest (ridit score of 0) values of the distribution of each socioeconomic characteristic. SII for urban/rural were not estimated, due to collinearity between the ridit score and the region covariate. GLM with Negative Binomial distribution and log link function were used. Fully adjusted models included adjustments for demographic, socioeconomic, lifestyle, and morbidity factors and region. Rates were standardised for the overall CKB participant population in 2009 for education and HI, and for the separate populations of CKB participants living in urban or rural areas, respectively, in 2009 for urban/rural. Exclusions as in figure 1.

webtable 4: Absolute differences in fully adjusted 28-day case fatality rates between lowest and highest category of each socioeconomic characteristic and slope index of inequality (SII) (per 100 events), by calendar year

	Stroke					Ischaemic heart disease				
	Urban/Rural	Education		HI		Urban/Rural	Education		HI	
	Diff[1]	Diff[1]	SII[2]	Diff[1]	SII[2]	Diff[1]	Diff[1]	SII[2]	Diff[1]	SII[2]
**2009**	14.30 (0.88)	0.03 (1.89)	0.86 (1.66)	7.04 (1.13)	16.05 (2.28)	11.73 (1.12)	4.03 (1.77)	4.43 (1.75)	13.16 (1.10)	16.05 (2.28)
**2010**	12.85 (0.90)	-0.40 (1.67)	1.92 (1.62)	3.80 (1.12)	14.61 (2.16)	10.75 (1.06)	6.79 (1.79)	8.46 (1.82)	12.57 (1.05)	14.61 (2.16)
**2011**	15.74 (0.88)	4.93 (1.70)	6.18 (1.51)	6.69 (1.08)	14.80 (2.12)	10.61 (1.04)	7.24 (1.65)	8.86 (1.69)	12.65 (1.03)	14.80 (2.12)
**2012**	14.97 (0.82)	2.79 (1.50)	4.67 (1.39)	6.26 (0.97)	9.38 (1.60)	8.82 (0.85)	-0.29 (1.41)	-0.37 (1.38)	9.62 (0.83)	9.38 (1.60)
**2013**	12.05 (0.77)	1.34 (1.40)	1.88 (1.28)	3.44 (0.91)	8.69 (1.44)	9.74 (0.77)	3.50 (1.18)	4.35 (1.19)	8.85 (0.77)	8.69 (1.44)
**2014**	11.75 (0.81)	-0.20 (1.51)	0.40 (1.39)	3.41 (0.93)	7.07 (1.42)	7.70 (0.78)	0.14 (1.27)	0.19 (1.26)	8.37 (0.77)	7.07 (1.42)
**2015**	10.05 (0.76)	-1.31 (1.34)	-1.08 (1.25)	3.02 (0.85)	7.49 (1.42)	5.70 (0.77)	3.11 (1.16)	3.67 (1.17)	8.30 (0.75)	7.49 (1.42)
**2016**	7.75 (0.75)	-2.21 (1.24)	-2.84 (1.18)	1.33 (0.82)	4.09 (1.20)	4.86 (0.72)	-0.44 (1.11)	-0.44 (1.10)	6.24 (0.69)	4.09 (1.20)
**p-value for trend**	<0.0001	0.0163	<0.0001	<0.0001	<0.0001	0.0022	0.0006	<0.0001	<0.0001	<0.0001

[1]Diff: absolute difference in predicted rates between the lowest and highest group of each socioeconomic characteristic. [2]SII: absolute difference in predicted rates between the lowest (ridit score of 0) and the highest (ridit score of 1) values of the distribution of each socioeconomic characteristic. SII for urban/rural were not estimated, due to collinearity between the ridit score and the region covariate. GLM with Binomial distribution and logit link function were used. Fully adjusted models included adjustments for demographic, socioeconomic, lifestyle, and morbidity factors, region and stroke or IHD type. Rates were standardised for the overall CKB participant population in 2009 for education and HI, and for the separate populations of CKB participants living in urban or rural areas, respectively, in 2009 for urban/rural. Exclusions as in figure 4.

webtable 5: Absolute differences in fully adjusted mean length of stay between highest and lowest category of each socioeconomic characteristic and slope index of inequality (SII) (in days), by calendar year

	Stroke					Ischaemic heart disease				
	Urban/Rural	Education		HI		Urban/Rural	Education		HI	
	Diff[1]	Diff[1]	SII[2]	Diff[1]	SII[2]	Diff[1]	Diff[1]	SII[2]	Diff[1]	SII[2]
**2009**	2.28 (0.35)	0.73 (0.73)	1.07 (0.65)	3.55 (0.36)	7.21 (0.61)	3.72 (0.80)	0.64 (0.59)	0.85 (0.74)	3.70 (0.77)	3.45 (1.34)
**2010**	2.74 (0.24)	1.84 (0.38)	2.38 (0.40)	3.81 (0.28)	7.75 (0.51)	4.45 (0.40)	0.96 (0.94)	1.44 (0.79)	4.40 (0.39)	5.00 (0.69)
**2011**	2.23 (0.28)	1.62 (0.38)	2.07 (0.41)	3.84 (0.31)	7.81 (0.56)	3.97 (0.23)	1.79 (0.39)	2.44 (0.40)	3.97 (0.23)	4.25 (0.48)
**2012**	1.51 (0.22)	1.32 (0.37)	1.71 (0.36)	3.04 (0.25)	6.15 (0.45)	2.58 (0.18)	0.73 (0.30)	0.94 (0.31)	1.99 (0.18)	0.62 (0.38)
**2013**	0.89 (0.24)	0.21 (0.45)	0.37 (0.43)	2.96 (0.29)	6.00 (0.51)	3.02 (0.25)	0.02 (0.54)	0.16 (0.49)	3.48 (0.25)	3.41 (0.49)
**2014**	1.07 (0.25)	0.18 (0.42)	0.27 (0.42)	2.40 (0.29)	4.84 (0.50)	3.68 (0.19)	-0.19 (0.37)	-0.18 (0.35)	3.74 (0.20)	4.02 (0.42)
**2015**	1.04 (0.21)	-1.05 (0.40)	-1.23 (0.37)	2.41 (0.25)	4.86 (0.45)	3.14 (0.16)	-0.33 (0.31)	-0.29 (0.29)	3.20 (0.17)	3.08 (0.35)
**2016**	1.01 (0.19)	-1.07 (0.33)	-1.39 (0.32)	2.04 (0.22)	4.10 (0.37)	1.94 (0.28)	-0.50 (0.33)	-0.85 (0.38)	-0.03 (0.17)	0.71 (0.49)
**p-value for trend**	<0.0001	<0.0001	<0.0001	<0.0001	<0.0001	0.0005	<0.0001	<0.0001	0.0609	0.0160

[1]Diff: absolute difference in predicted mean LOS between the highest and lowest group of each socioeconomic characteristic. [2]SII: absolute difference in predicted mean LOS between the highest (ridit score of 1) and the lowest (ridit score of 0) values of the distribution of each socioeconomic characteristic. SII for urban/rural were not estimated, due to collinearity between the ridit score and the region covariate. GLM with Gamma distribution and log link function were used. Fully adjusted models included adjustments for demographic, socioeconomic, lifestyle, and morbidity factors, region and stroke or IHD type. Mean LOS were standardised for the overall CKB participant population in 2009 for education and HI, and for the separate populations of CKB participants living in urban or rural areas, respectively, in 2009 for urban/rural. Exclusions as in figure 5.

webtable 6: Absolute annual differences in adjusted rates of hospitalisation, 28-day case fatality rates, mean length of hospital stay and slope index of inequality (SII) between rural and urban participants, without region adjustment

	Rates of hospitalisation (per 1000 person-years)				Case fatality rates (per 100 events)				Mean length of stay (in days)			
	Stroke		Ischaemic heart disease		Stroke		Ischaemic heart disease		Stroke		Ischaemic heart disease	
	Diff[1]	SII[2]	Diff[1]	SII[2]	Diff[1]	SII[2]	Diff[1]	SII[2]	Diff[1]	SII[2]	Diff[1]	SII[2]
**2009**	1.34 (0.33)	2.68 (0.52)	0.71 (0.34)	1.42 (0.53)	3.49 (1.05)	70.13 (16.46)	3.42 (1.20)	68.78 (19.24)	2.04 (0.39)	4.14 (0.62)	2.23 (1.39)	4.54 (2.16)
**2010**	0.86 (0.33)	1.72 (0.52)	0.91 (0.34)	1.81 (0.54)	1.36 (1.04)	27.21 (16.41)	2.03 (1.10)	40.61 (17.59)	2.80 (0.27)	5.71 (0.46)	3.54 (0.51)	7.33 (0.81)
**2011**	3.00 (0.32)	5.97 (0.51)	2.25 (0.33)	4.47 (0.53)	4.85 (1.03)	97.4 (16.16)	2.21 (1.07)	44.30 (17.16)	1.98 (0.32)	4.01 (0.53)	3.05 (0.26)	6.30 (0.45)
**2012**	2.57 (0.33)	5.11 (0.53)	-1.88 (0.38)	-3.82 (0.64)	4.49 (0.93)	90.15 (14.66)	1.57 (0.88)	31.39 (13.96)	0.86 (0.23)	1.73 (0.37)	1.70 (0.20)	3.46 (0.34)
**2013**	3.17 (0.34)	6.31 (0.54)	-1.20 (0.35)	-2.43 (0.58)	2.22 (0.87)	44.47 (13.67)	2.60 (0.8)	52.23 (12.84)	0.53 (0.29)	1.06 (0.47)	1.98 (0.28)	4.02 (0.48)
**2014**	1.33 (0.32)	2.64 (0.50)	-2.51 (0.34)	-5.09 (0.58)	2.02 (0.90)	40.48 (14.02)	0.32 (0.79)	6.42 (12.40)	0.91 (0.28)	1.83 (0.46)	3.03 (0.24)	6.25 (0.45)
**2015**	2.17 (0.30)	4.32 (0.47)	-0.18 (0.31)	-0.36 (0.50)	1.34 (0.86)	26.79 (13.43)	-0.85 (0.78)	-17.01 (12.34)	0.88 (0.25)	1.77 (0.41)	2.35 (0.20)	4.82 (0.36)
**2016**	1.00 (0.31)	1.98 (0.48)	-3.52 (0.33)	-7.20 (0.56)	-0.81 (0.88)	-16.21 (13.56)	-1.68 (0.72)	-33.64 (11.45)	1.23 (0.25)	2.48 (0.42)	1.64 (0.29)	3.33 (0.48)
**p-value for trend**	0.772	0.6832	<0.0001	<0.0001	0.0003	<0.0001	<0.0001	<0.0001	<0.0001	<0.0001	0.0984	0.0161

[1]Diff: absolute difference in predicted rates of hospitalisation/mean LOS (or case fatality rates) between the highest (lowest) and lowest (highest) group of each socioeconomic characteristic. [2]SII: absolute difference in predicted rates of hospitalisation/mean LOS (case fatality rates) between the highest (lowest) and the lowest (highest) values of the distribution of each socioeconomic characteristic. For rates of hospitalisation: GLM with Negative Binomial distribution and log link were used. For case fatality rates: GLM with Binomial distribution and logit link function were used. For mean LOS: GLM with Gamma distribution and log link function were used. Fully adjusted models included adjustments for demographic, socioeconomic, lifestyle, and morbidity factors (and stroke or IHD type for case fatality rates and mean LOS). Rates/mean LOS were standardised for the overall CKB participant population in 2009. Exclusions as in webtables 3-5.

webtable 7: Annual percentage change in fully adjusted rates of hospitalisation, 28-day case fatality and mean length of stay for stroke, ischaemic heart disease and any cause, by age group

	Rates of hospitalisation		Case fatality rates		Mean length of stay	
	**Stroke**	**Ischaemic heart disease**	**Stroke**	**Ischaemic heart disease**	**Stroke**	**Ischaemic heart disease**
**<60 years old**	3.73 (2.91, 4.56)	5.78 (4.83, 6.74)	-6.53 (-10.06, -2.86)	-10.35 (-14.14, -6.39)	-2.01 (-2.61, -1.40)	-2.18 (-3.16, -1.20)
**60-70 years old**	4.29 (3.61, 4.98)	6.17 (5.36, 6.98)	-13.95 (-16.84, -10.95)	-15.95 (-19.09, -12.69)	-1.84 (-2.28, -1.39)	-2.14 (-3.47, -0.79)
**≥70 years old**	2.95 (2.28, 3.64)	4.58 (3.78, 5.39)	-13.11 (-15.53, -10.63)	-14.98 (-17.47, -12.42)	-1.78 (-2.25, -1.31)	-0.84 (-1.51, -0.16)

For rates of hospitalisation: GLM with Negative Binomial distribution and log link function were used. For 28-day case fatality rates, GLM with Binomial distribution and logit link function were used. For mean LOS: GLM with Gamma distribution and log link function were used. Fully adjusted models included adjustments for demographic, socioeconomic, lifestyle, and morbidity factors, and region (and stroke and IHD type for mean LOS and case fatality rates). Exclusions as in figure 3.

webfigure 1: Annual unadjusted and fully adjusted rates of hospitalisation for stroke, ischaemic heart disease and any cause

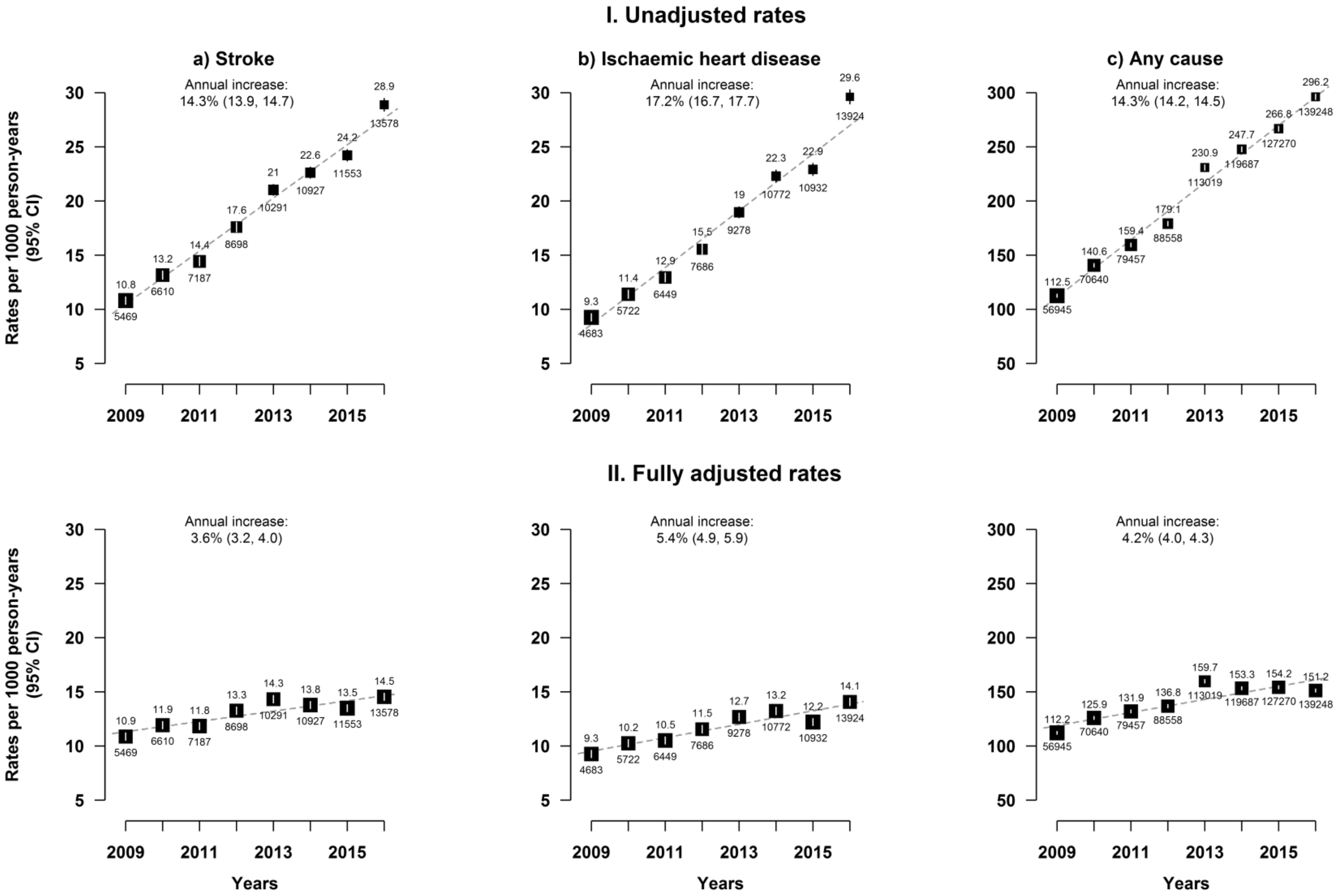

GLM with Negative Binomial distribution and log link function were used. Fully adjusted models included adjustment for demographic, socioeconomic, lifestyle, and morbidity factors, and region. Rates of hospitalisation were standardised for the overall CKB participant population in 2009. Numbers above the squares are rates per 1000 person-years. Numbers below the squares are numbers of admissions. The area of each square is inversely proportional to the variance. Exclusions as in figure 1.

webfigure 2: Fully adjusted 28-day case fatality rates for stroke and ischaemic heart disease, by calendar year

GLM with Binomial distribution and logit link function were used. Rates were adjusted for demographic, socioeconomic, lifestyle, and morbidity factors, region and stroke or IHD type as appropriate, and standardised for the CKB participant population in 2009. Exclusions as in figure 4. Numbers above the squares are 28-day case fatality rates per 100 events and numbers below the squares are number of cases. Exclusions as in figure 4.

webfigure 3: Fully adjusted annual rates of hospitalisation, 28-day case fatality and mean length of stay for stroke and ischaemic heart disease, by income

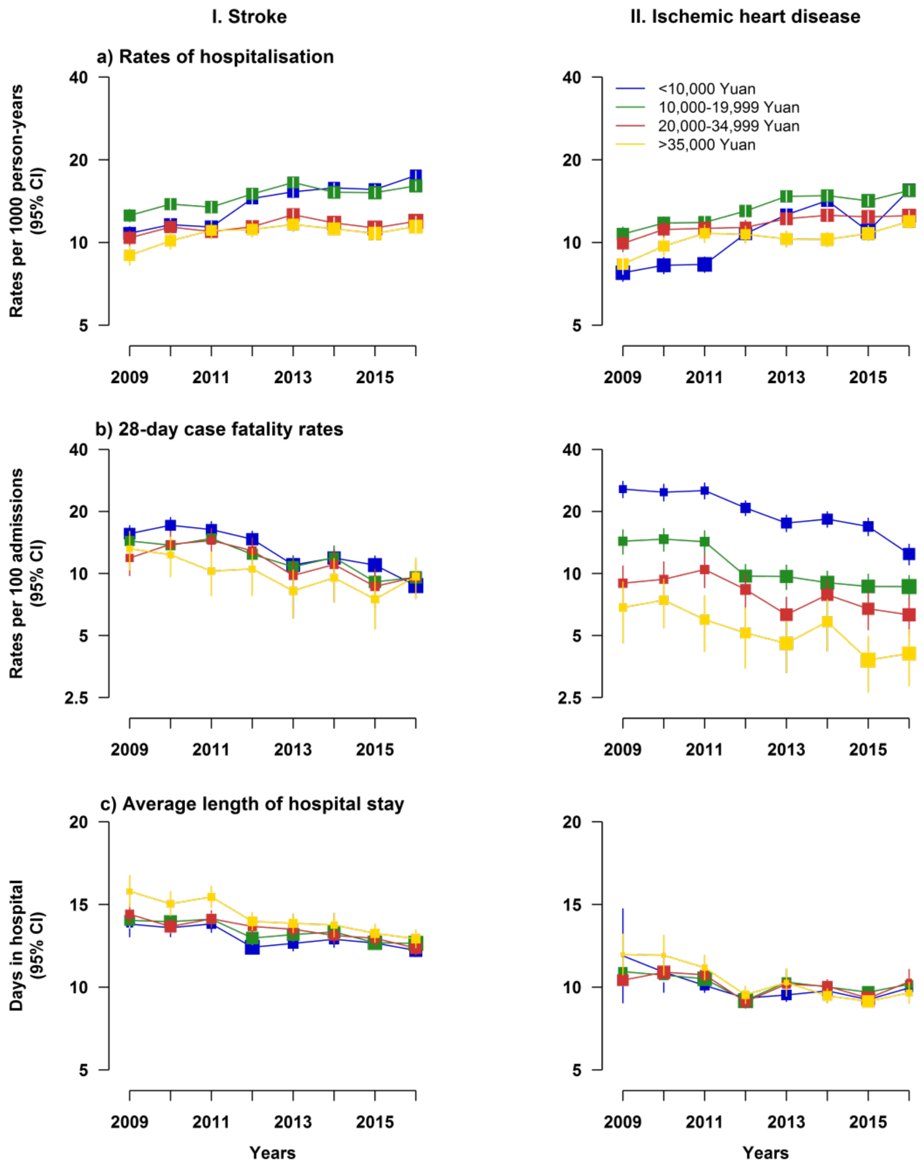

For rates of hospitalisation, GLM with Negative Binomial distribution and log link function were used. For 28-day case fatality rates, GLM with Binomial distribution and logit link function were used. For mean LOS, GLM with Gamma distribution and log link function were used. Fully adjusted models included adjustment for demographic, socioeconomic, lifestyle, and morbidity factors, and region (and stroke or IHD type for case fatality and mean LOS). All estimates were standardised for the overall CKB participant population in 2009. Exclusions as in figure 3.

webfigure 4: Fully adjusted annual mean length of hospital stay for stroke and ischaemic heart disease, by urban and rural area, education and health insurance type

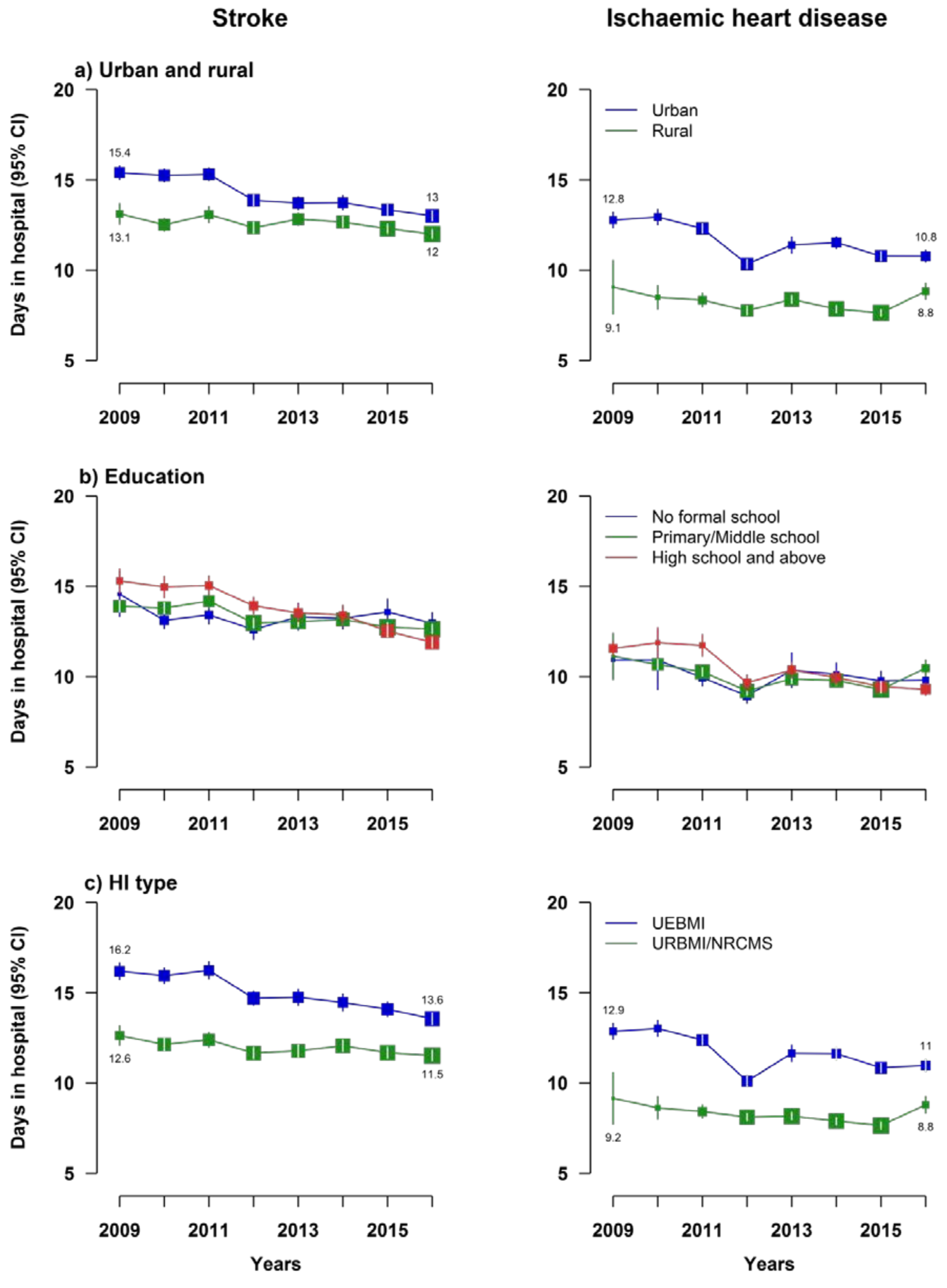

GLM with Gamma distribution and log link function were used. Fully adjusted models included adjustment for demographic, socioeconomic, lifestyle, and morbidity factors, region and stroke or IHD type as appropriate. In analyses by HI type, uninsured participants were excluded due to small number of events. Mean LOS were standardised for the overall CKB participant population in 2009 for education and HI type, and for the separate populations of CKB participants living in urban or rural areas, respectively, in 2009 for urban and rural area. Numbers above the squares are mean LOS in days. The area of each square is inversely proportional to the variance.

webfigure 5: Fully adjusted annual percentage change in rates of hospitalisation and mean length of hospital stay for any cause, by urban and rural area, education, income and health insurance type

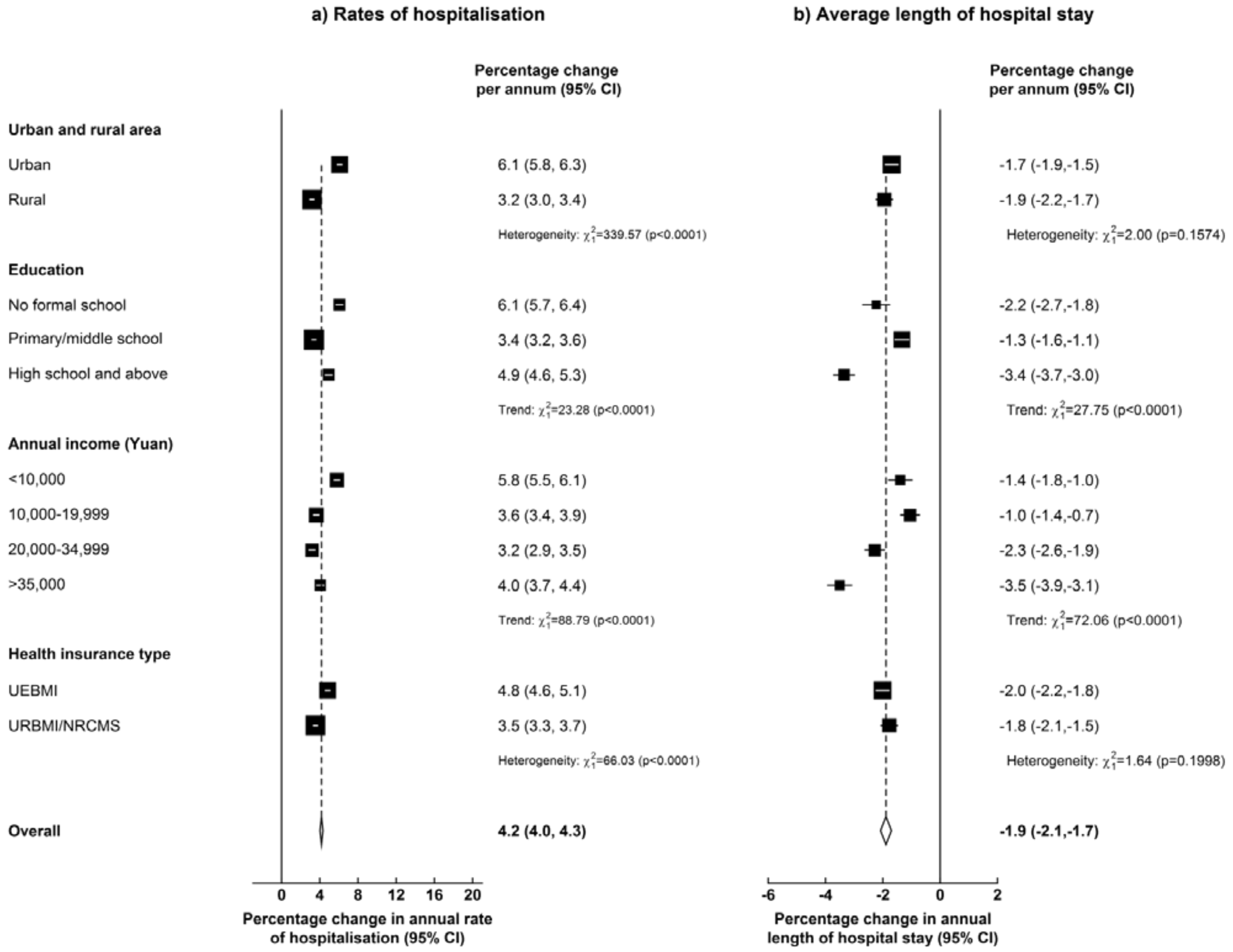

For rates of hospitalisation: GLM with Negative Binomial distribution and log link function were used. For mean LOS: GLM with Gamma distribution and log link function were used. Fully adjusted models were adjusted for demographic, socioeconomic, lifestyle, and morbidity factors, and region. Models for mean LOS were also adjusted for stroke and IHD type, as appropriate. In analyses by HI type, uninsured participants were excluded due to small number of events. UEBMI: Urban Employee Basic Medical Insurance. URBMI/NRCMS: Urban Resident Basic Medical Insurance/New Rural Cooperative Medical Scheme. The area of each square is inversely proportional to the variance. Exclusions as in figures 1 and 5.

webfigure 6: Fully adjusted rates of hospitalisation and mean length of hospital stay for first-ever hospital admission for stroke and ischaemic heart disease

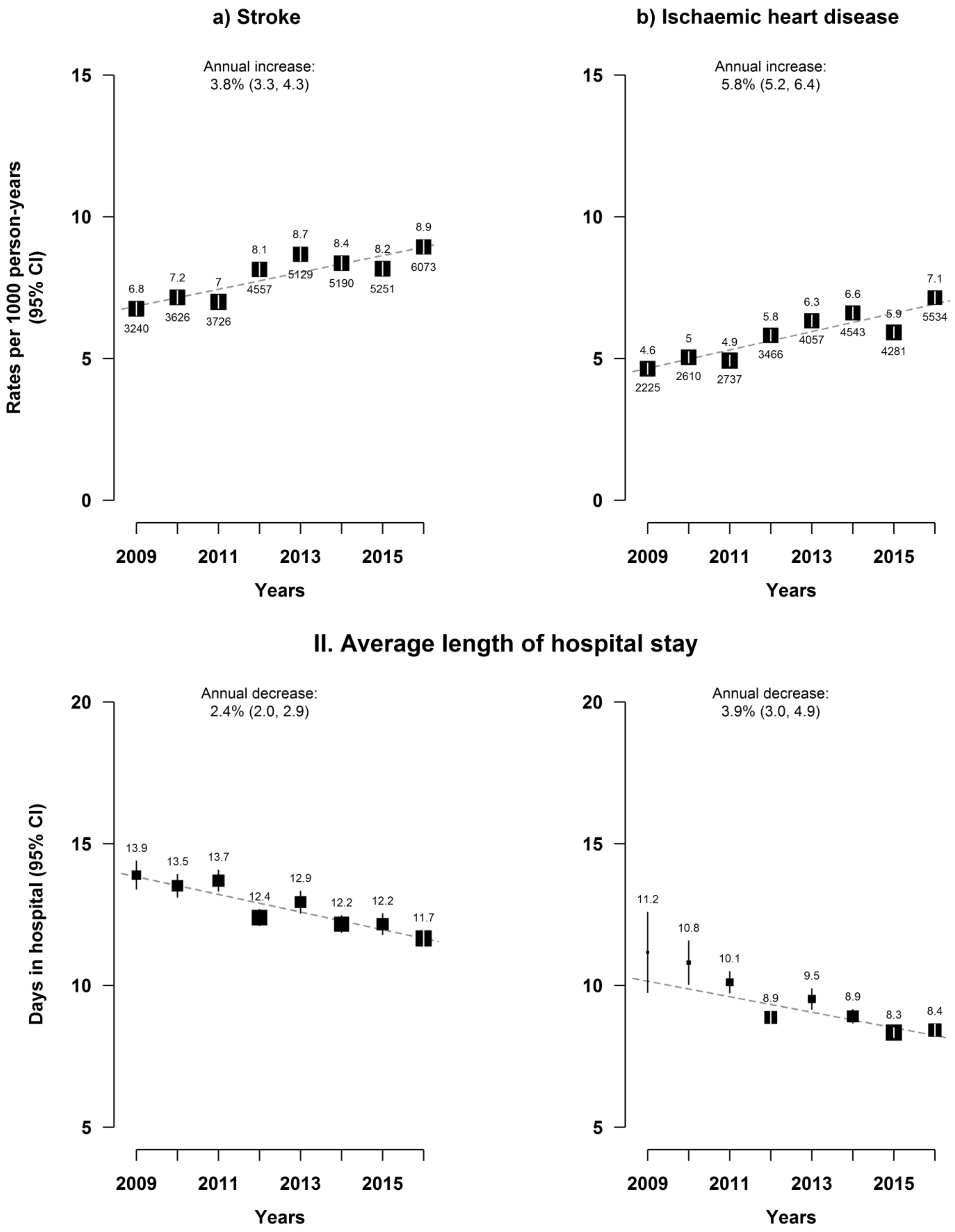

For rates of hospitalisation, models used were GLM with Negative Binomial distribution and log link function. For mean LOS, models used were GLM with Gamma distribution and log link function. Fully adjusted models were adjusted for demographic, socioeconomic, lifestyle, and morbidity factors, and region (and stroke or IHD type for mean LOS). Rates of hospitalisation and mean LOS were standardised for the overall CKB participant population in 2009. Numbers above the squares are rates per 1000 person-years or mean length of stay in days, as appropriate. Numbers below the squares are numbers of admissions. The area of each square is inversely proportional to the variance. Exclusions as in figure 1 for models for rate of hospitalisation.

webfigure 7: Fully adjusted rates of hospitalisation and mean length of hospital stay, by type of stroke and ischaemic heart disease

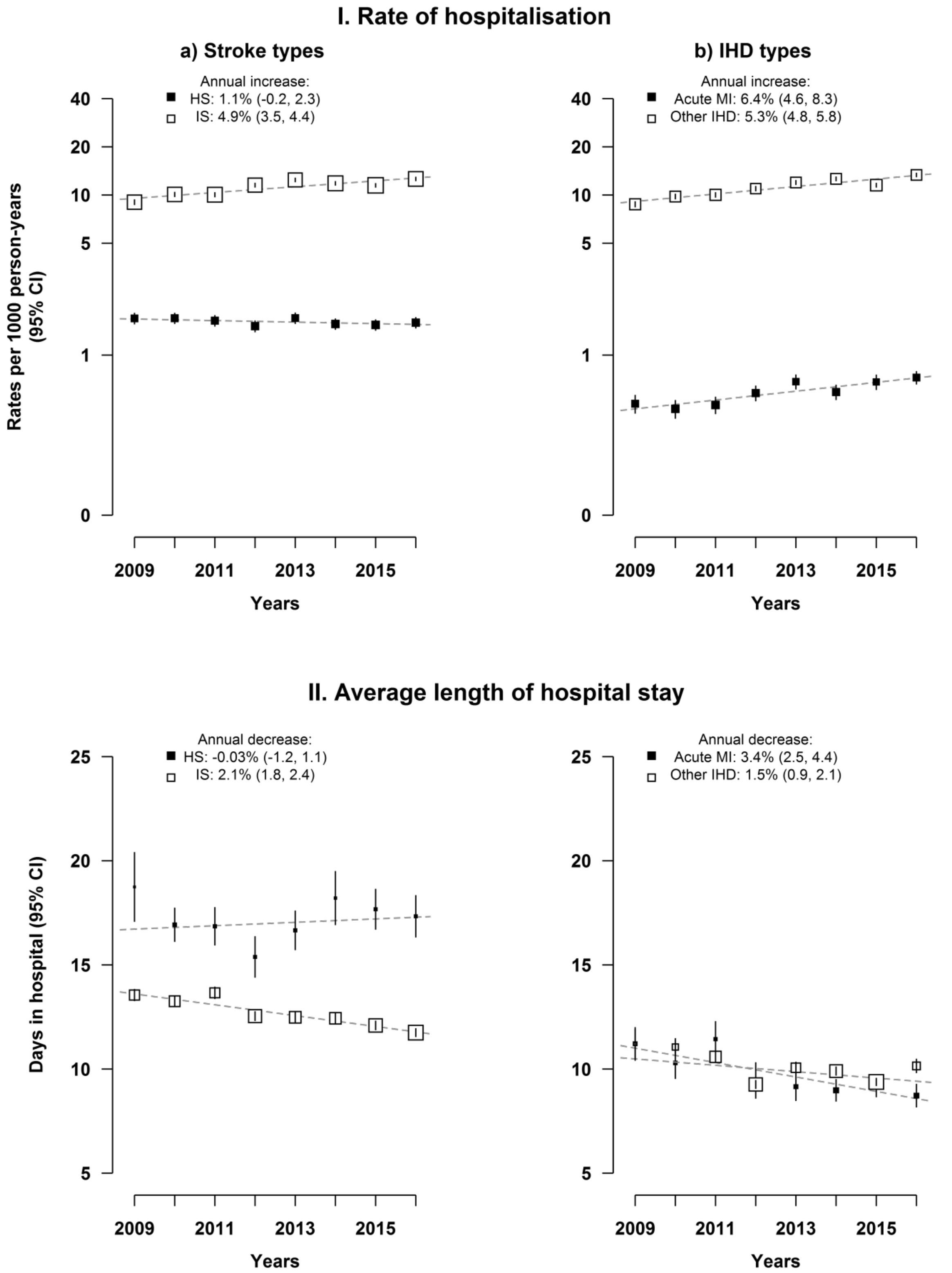

For rates of hospitalisation, models used were GLM with Negative Binomial distribution and log link function. For mean LOS, models used were GLM with Gamma distribution and log link function. Fully adjusted models were adjusted for demographic, socioeconomic, lifestyle, and morbidity factors, and region (and stroke or IHD type for mean LOS). Rates of hospitalisation and mean LOS were standardised for the overall CKB participant population in 2009. Exclusions as in figure 1 and 5.

webfigure 8: Fully adjusted rates of hospitalisation, 28-day case fatality rates and mean length of hospital stay for stroke and ischemic heart disease, by age group

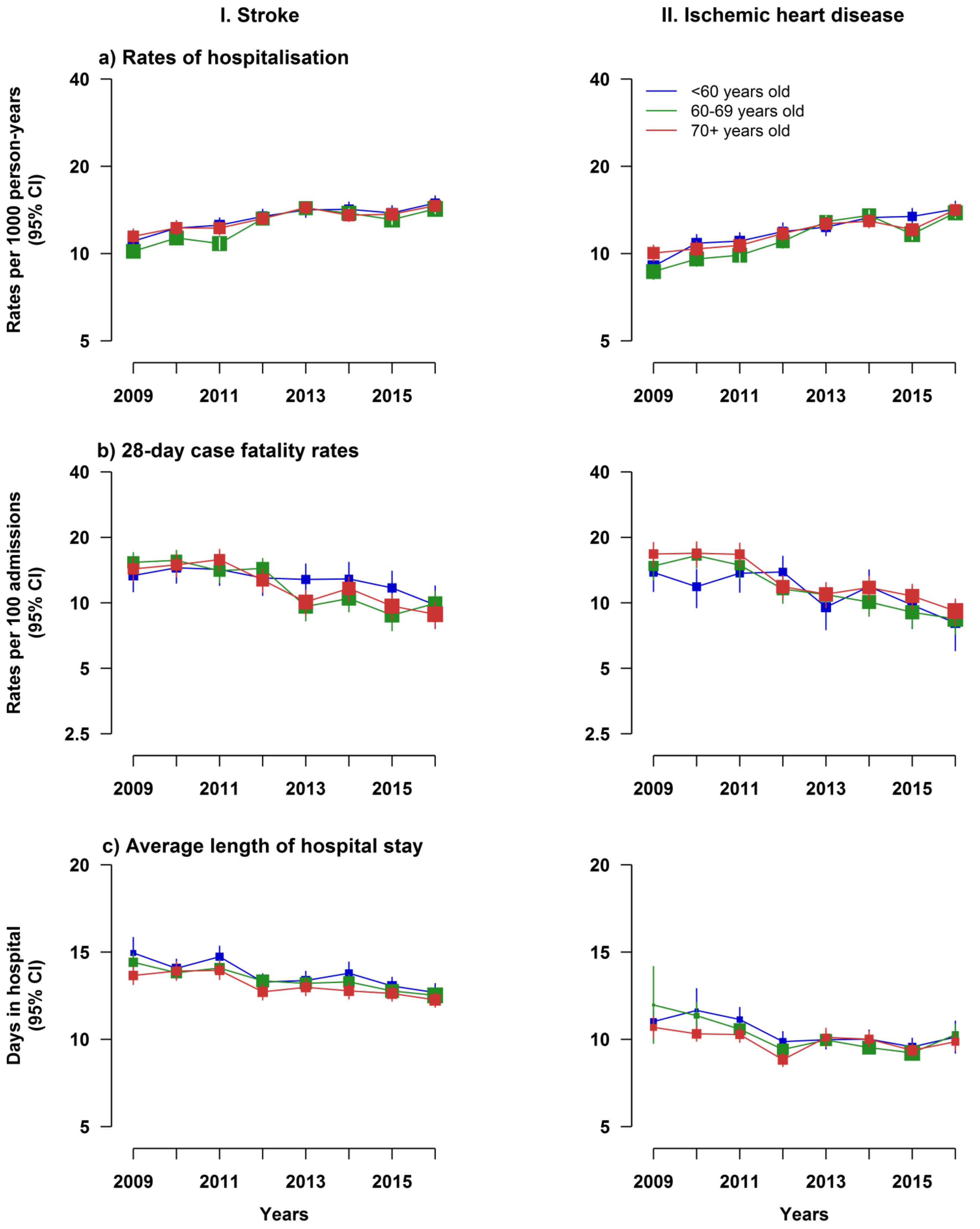

For rates of hospitalisation, models used were GLM with Negative Binomial distribution and log link function. For case fatality rates, models used were GLM with Binomial distribution and logit link function. For mean LOS, models used were GLM with Gamma distribution and log link function. Fully adjusted models were adjusted for demographic, socioeconomic, lifestyle, and morbidity factors, and region (and stroke or IHD type for case fatality rates and mean LOS). All estimates were standardised for the overall CKB participant population in 2009. Exclusions as in figure 3.
